# Panomics Integration via Machine Learning Prioritizes TAF1D as a Therapeutic Vulnerability in Lung Adenocarcinoma

**DOI:** 10.1155/humu/1816649

**Published:** 2026-04-11

**Authors:** Lan Ding, Qingmei Xu, Dongdong Liu, Jingyu Wu, Xufan Cai, Feiqi Xu, Shuhan Ma, Haitao Wang, Yanyan Shi

**Affiliations:** ^1^ Department of Thoracic Surgery, Cancer Center, Zhejiang Provincial People′s Hospital, Affiliated People′s Hospital, Hangzhou Medical College, Hangzhou, Zhejiang, China, hznu.edu.cn; ^2^ Department of Nursing, Zhejiang Provincial People′s Hospital, Affiliated People′s Hospital, Hangzhou Medical College, Hangzhou, Zhejiang, China, hznu.edu.cn; ^3^ Geriatric Ward, The 903rd Hospital of the Joint Logistics Support Force of the Chinese People′s Liberation Army, Hangzhou, Zhejiang, China; ^4^ Department of General Surgery, Zhejiang Cancer Hospital, Hangzhou, Zhejiang, China, zchospital.com; ^5^ Department of Graduate, Hangzhou Normal University, Hangzhou, Zhejiang, China, hznu.edu.cn

**Keywords:** cell proliferation, immune cell infiltration, lung adenocarcinoma, SHAP analysis, single-cell RNA sequencing, somatic mutation, TAF1D

## Abstract

Lung adenocarcinoma (LUAD) is a leading cause of cancer mortality, necessitating the identification of robust biomarkers and a deeper understanding of its molecular underpinnings. This study is aimed at screening for potential LUAD biomarkers and characterizing their biological functions. Using an integrative computational framework, we combined multitranscriptomic data analysis with three machine learning algorithms (LASSO, SVM‐RFE, and random forest) to identify a consensus seven‐gene signature (TTC13, TAF1D, ZNF587, PRPF3, LINC01355, TARBP1, and CCNL2). A classifier based on this signature achieved exceptional diagnostic accuracy (AUC = 0.972), with TAF1D identified as the most influential predictor via SHAP analysis. TAF1D was significantly upregulated in tumors, correlated with an immunosuppressive microenvironment, and promoted cancer cell proliferation by regulating cell cycle and immune‐related pathways. Critically, TAF1D exhibited significant spatial heterogeneity in expression across different samples and tissue regions, suggesting it may exert region‐specific biological functions within the tumor. In conclusion, our work defines a validated gene signature for LUAD, nominating TAF1D as a key oncogenic driver and promising candidate for diagnostic and therapeutic development.

## 1. Introduction

Lung adenocarcinoma (LUAD) is a common malignant tumor and a major histological subtype of non–small cell lung cancer (NSCLC). Its incidence is on the rise globally, and it has become the most common type of lung cancer, particularly among never‐smokers [1–5]. The diagnosis of LUAD mainly relies on histopathological examinations and imaging examinations [2, 6]. Deep learning models have been trained to accurately and automatically classify and predict mutations in LUAD from histopathological images [6]. Computed tomography (CT) is an important imaging tool for lung cancer diagnosis; however, manual analysis of CT images is time‐consuming and error‐prone. Therefore, the development of new diagnostic models is conducive to the accurate diagnosis of LUAD.

In previous studies, numerous diagnostic models for LUAD have been developed [7]. Nevertheless, due to their complex internal structures and nonlinear transformations, machine learning models often lack inherent interpretability and are referred to as “black‐box” models [8–10]. Traditional research on machine learning primarily focuses on model prediction accuracy, generalization ability, and efficiency [11]. Its core goal is to achieve high predictive performance or classification accuracy, while its internal decision‐making process is difficult for humans to understand [8]. In contrast, the SHAP (SHapley Additive exPlanations) algorithm is a model‐agnostic post hoc interpretability technique. Its main purpose is to provide interpretability for the predictions of machine learning models and reveal the contribution of each feature to the model output [9, 12, 13]. The SHAP algorithm does not replace pure machine learning models but rather complements their functionality. After model training is completed, it provides explanations for model predictions (post hoc explanation) [10]. Many studies have combined high‐performance machine learning models (e.g., XGBoost and deep learning models) with SHAP to develop systems that not only achieve high predictive performance but also possess interpretability [14–22]. For instance, in genomic prediction, the combination of automated machine learning (AutoML) with SHAP can improve prediction stability and enhance model transparency [16]. In brain tumor diagnosis, the integration of principal component analysis (PCA), support vector classifier (SVC), and SHAP aims to achieve optimal performance and interpretability [17]. In our study, we used the SHAP algorithm to identify the most critical factors for the LUAD diagnostic model, addressing the limitation of machine learning models in terms of interpretability.

This study not only identifies a set of potential LUAD biomarkers but also clarifies the role of TAF1D as a key regulator of cell proliferation and immune‐related pathways, thereby providing a theoretical foundation for the development of novel diagnostic tools and therapeutic targets for LUAD.

## 2. Methods

### 2.1. Data Collection

Transcriptomic data of LUAD were obtained from the UCSC Xena publication page (https://xena.ucsc.edu/) and the GEO (Gene Expression Omnibus) (https://www.ncbi.nlm.nih.gov/geo/query) databases. The UCSC Xena and GEO datasets were merged, and batch effects were removed via batch correction methods to ensure data consistency. The LUAD cohorts included in our study met the following criteria: (1) pathologically confirmed diagnosis of LUAD, (2) availability of complete transcriptome expression data, and (3) sufficient sample size for statistical analysis.

### 2.2. Differential Expression and Functional Enrichment Analysis

Differential expression analysis was performed using the limma R package (V4.0.0). A linear model was fitted using lmFit and eBayes based on an intercept‐free design matrix. Differentially expressed genes (DEGs) were identified using the following criteria: |log2*F* *C*| > 0.585 (1.5‐fold change) and *P*_FDR_ < 0.05. Functional enrichment for Gene Ontology (GO) and Kyoto Encyclopedia of Genes and Genomes (KEGG) pathways was conducted via the clusterProfiler package, with significance defined as *p* < 0.05 and *q* < 0.05.

### 2.3. Weighted Gene Coexpression Network Analysis (WGCNA)

A scale‐free coexpression network was constructed using the WGCNA package. The optimal soft‐thresholding power was determined where the scale‐free topology fit index reached 0.8. A topological overlap matrix (TOM) was generated to identify modules via dynamic tree cutting. Modules with high similarity were merged. Module membership (MM) and gene significance (GS) were calculated to evaluate the correlation between modules and clinical traits.

### 2.4. Machine Learning–Based Feature Selection

Three machine learning algorithms were employed to screen for feature genes: SVM‐RFE: We used a linear kernel function (kernel = “linear”) and set the penalty coefficient cost = 10 to obtain feature weights. Random forest (RF): Features were selected based on a Mean Decrease Gini score > 10, using an optimized number of trees determined by minimum error. LASSO regression: A binomial LASSO model was constructed via glmnet (10‐fold cross‐validation).

### 2.5. Model Evaluation and SHAP Interpretation

The optimal model was selected based on the area under the curve (AUC). To interpret the model, SHAP values were computed using permshap to rank genes by their contribution to the predicted probability.

### 2.6. Single‐Cell and Spatial Transcriptomic Analysis

Single‐cell RNA‐seq (scRNA‐seq) data were processed using Seurat. Following normalization and PCA, clusters were identified via t‐SNE and annotated using SingleR. For spatial transcriptomics, data were normalized via SCTransform. Dimensionality reduction and clustering were performed using PCA and UMAP to visualize gene spatial distribution.

### 2.7. In Silico Gene Knockout via scTenifoldKnk

Virtual knockout of TAF1D was simulated using scTenifoldKnk. The pipeline involved constructing 10 multilayer networks (500 cells per network) and reducing dimensions to three components to calculate the differential regulation fold change of downstream genes.

### 2.8. Correlation Analysis Between TAF1D and Clinical Features

Online databases were used to analyze the differential expression of TAF1D and its associations with tumor stage, gender, race, age, and prognosis.

### 2.9. Somatic Mutation Analysis

Somatic mutation analysis of TAF1D in LUAD was performed using the cBioPortal platform based on the TCGA Pan‐Cancer Atlas cohort. The dataset includes genomic and clinical information from TCGA‐LUAD patients. Genetic alterations including missense mutations, truncating mutations, copy number amplifications, and deep deletions were analyzed using the OncoPrint module. Mutation distribution across the protein sequence was assessed using the mutation mapper tool. Survival analysis was conducted using the Kaplan–Meier method with the log‐rank test to evaluate the association between TAF1D alterations and overall survival. Hazard ratios (HRs) were calculated using the built‐in survival analysis module. Comutation analysis was performed using the comparison module to identify significantly enriched mutated genes in TAF1D‐altered versus unaltered groups.

### 2.10. Gene Set Enrichment Analysis (GSEA)

To investigate the biological pathways associated with TAF1D, samples were stratified into high‐ and low‐expression groups based on the median expression level of the gene. A ranked gene list was generated by calculating the log_2_FC of mean expression between the two cohorts. GSEA was subsequently performed using the clusterProfiler package, referencing the KEGG gene sets. Pathways with a *p* < 0.05 were considered significantly enriched.

### 2.11. LUAD Cell Culture

Human LUAD cell lines (AEC, A549, and PC‐9) were used. The culture medium was RPMI‐1640 or DMEM supplemented with 10% fetal bovine serum (FBS), 100 U/mL penicillin, and 100 *μ*g/mL streptomycin (purchased from Gibco). Cells were cultured in a 37°C incubator with 5% CO_2_, and the medium was replaced every 2–3 days. When cell confluency reached 80%–90%, the medium was discarded, and cells were washed twice with PBS. Cells were digested with 0.25% trypsin for 1–2 min; digestion was terminated, and cells were pipetted into a single‐cell suspension, followed by passage at a 1:3 ratio.

### 2.12. Quantitative Real‐Time PCR (qPCR)

Total RNA was isolated from harvested digestive cells using a spin column method (Vazyme Biotech Co. Ltd, Nanjing, China). RNA purity and concentration were determined by UV spectrophotometry, with A260/A280 ratios between 1.8 and 2.0 indicating high purity. According to the manufacturer′s instructions (Vazyme Biotech Co. Ltd, Nanjing, China), RNA was reverse‐transcribed into cDNA. Quantitative PCR was performed using SYBR Green Premix (Vazyme Biotech Co. Ltd, Nanjing, China) on an Applied Biosystems 7500 Real‐Time PCR System. The thermal cycling conditions were as follows: initial denaturation at 95°C for 30 s, followed by 40 cycles of denaturation at 95°C for 5 s, and annealing/extension at 60°C for 30 s. The relative expression of the target genes was calculated using the 2^−*ΔΔ*Ct^ method, with three technical replicates per sample. GAPDH was used as the internal reference gene. The primer sequences designed for the target genes are listed as follows:

TAF1D‐F: CTCAGTGTATCCCTTACTCACCT

TAF1D‐R: CACTTGATGAATCACTTGCGTG

GAPDH‐F: GGAGCGAGATCCCTCCAAAAT

GAPDH‐R: GGCTGTTGTCATACTTCTCATGG

### 2.13. Modulation of TAF1D Expression

To modulate *TAF1D* levels, small interfering RNAs (siRNAs) targeting *TAF1D* and recombinant overexpression plasmids (pcDNA3.1(+)‐TAF1D), along with their respective negative controls, were synthesized by GeneChem (Shanghai, China). LUAD cells were transfected using Lipofectamine 3000 according to the manufacturer′s instructions. The sequences of the siRNA are listed as follows:

Si1: sense strand: 5 ^′^‐GAGUGUAUACUUAGAAGAATT‐3 ^′^

antisense strand: 5 ^′^‐UUCUUCUAAGUAUACACUCTT‐3 ^′^

Si2: sense strand: 5 ^′^‐CCUGAAAGUGUUCACGCAATT‐3 ^′^

antisense strand: 5 ^′^‐UUGCGUGAACACUUUCAGGTT‐3 ^′^

Si3: sense strand: 5 ^′^‐GCAAGCUGUUGCAAGAGGATT‐3 ^′^

antisense strand: 5 ^′^‐UCCUCUUGCAACAGCUUGCTT‐3 ^′^

### 2.14. Western Blot (WB)

Cells were harvested and lysed in RIPA lysis buffer supplemented with protease and phosphatase inhibitors. Protein concentrations were determined using a BCA protein assay kit. Equal amounts of protein extracts were separated by 10% SDS‐PAGE and transferred onto PVDF membranes. The membranes were blocked with 5% nonfat milk or BSA in TBST for 1 h at room temperature, then incubated overnight at 4°C with primary antibodies against TAF1D (1:1000 dilution) and GAPDH (1:100,000 dilution) as an internal control. After washing with TBST three times, the membranes were incubated with corresponding horseradish peroxidase (HRP)–conjugated secondary antibodies (1:4000 dilution) for 1 h at room temperature. Protein bands were visualized using an enhanced chemiluminescence (ECL) detection system. The relative expression levels of target proteins were normalized to the internal control.

### 2.15. CCK‐8 Assay

Log‐phase cells from the control group, knockdown group, and overexpression group were seeded into 96‐well plates at a density of 1 × 10^3^–5 × 10^3^ cells/well, with three replicate wells per group (the blank group contained only medium). At 0, 24, 48, and 72 h of culture, 10 *μ*L of CCK‐8 reagent (Dojindo) was added to each well, followed by incubation at 37°C for 2 h. The absorbance (OD value) at 450 nm was measured using a microplate reader (Bio‐Rad). Using the OD value at 0 h as the control, the relative proliferation rate at each time point was calculated as follows: Relative proliferation rate = (OD value of experimental group / OD value of control group) × 100%

### 2.16. Statistical Analysis

All statistical analyses in this study were performed using the R language (Version ≥ 4.0.0). The data were expressed as mean ± standard deviation (*x̅*±*s*), and the statistical significance level was set at *α* = 0.05 (*p* < 0.05 was considered statistically significant). The limma and clusterProfiler packages were used for differential expression analysis and enrichment analysis, respectively. Machine learning algorithms with 10‐fold cross‐validation were combined to screen for feature genes, and *t*‐test or analysis of variance (ANOVA) was used for comparison between groups. * p <0.05. ** p <0.01. *** p <0.001.

## 3. Results

### 3.1. Results of Batch Correction and Differential Analysis

**Figure 1:**
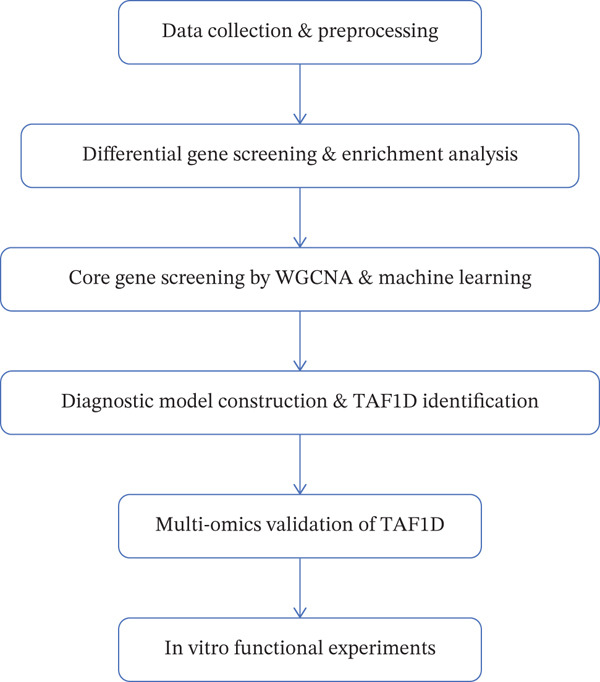
Workflow diagram.

The workflow diagram is shown in Figure 1. Before batch correction (Figure 2a), samples from different datasets were sparsely distributed in PCA; after batch correction (Figure 2b), the samples clustered closely, indicating that batch effects had been effectively eliminated. Subsequently, differential analysis was performed on the merged dataset. This study generated a heatmap to present the results (Figure 2c). Through this analytical workflow, a total of 4447 DEGs were detected, including 1841 upregulated genes and 2606 downregulated genes (Figure 2c). Another heatmap was generated in this study to further visualize relevant data (Figure 2d).

Figure 2Data difference analysis and functional analysis. (a) PCA plot of samples before batch correction. (b) PCA plot of samples after batch correction. (c) Volcano plot of differentially expressed genes (DEGs). (d) Heatmap of DEGs. (e) GO functional enrichment analysis plot. (f) KEGG functional enrichment analysis plot.

**Figure:**
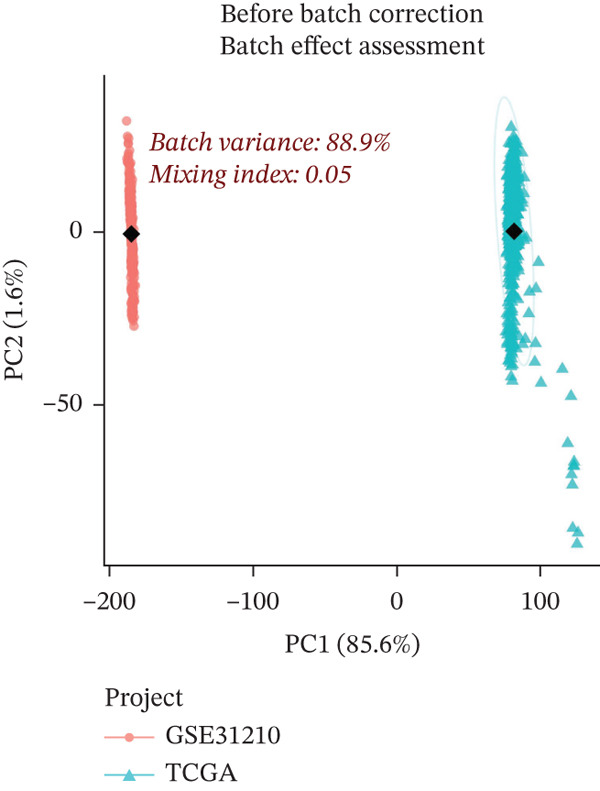
(a)

**Figure:**
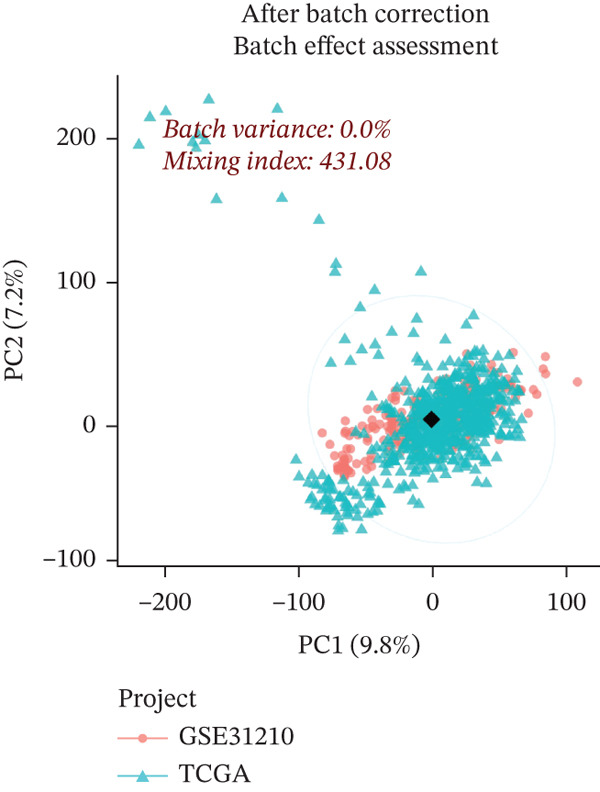
(b)

**Figure:**
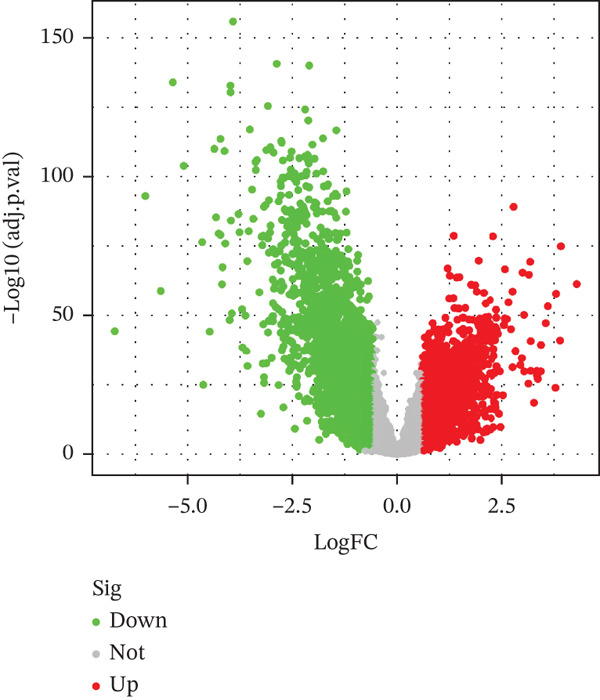
(c)

**Figure:**
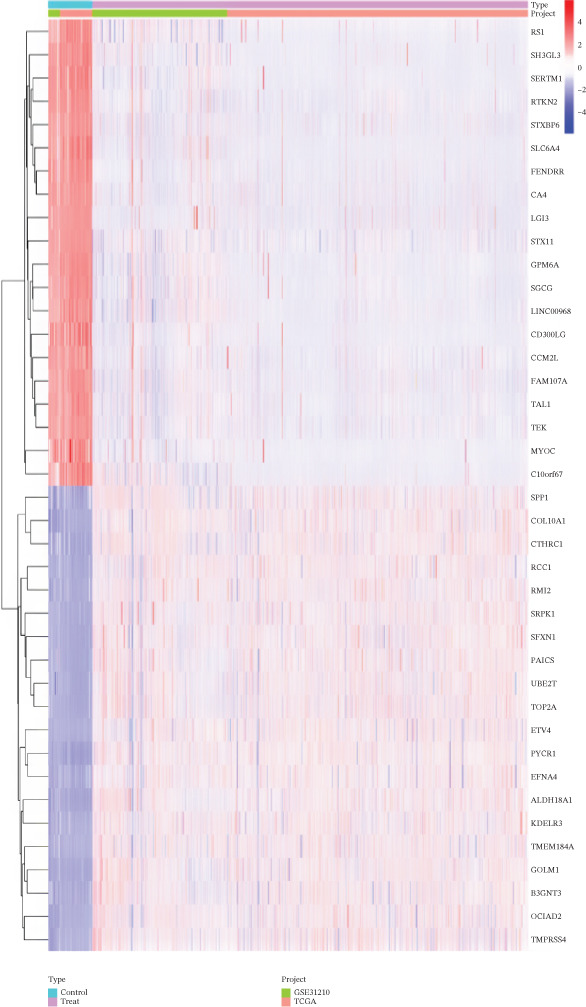
(d)

**Figure:**
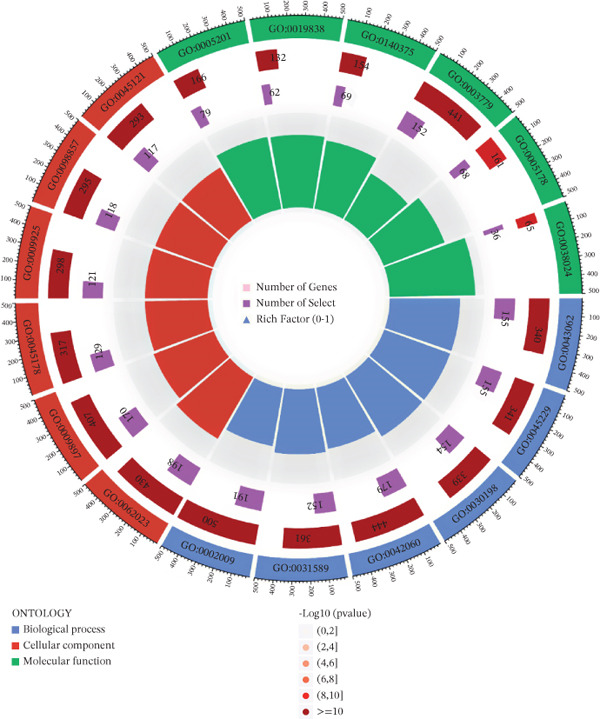
(e)

**Figure:**
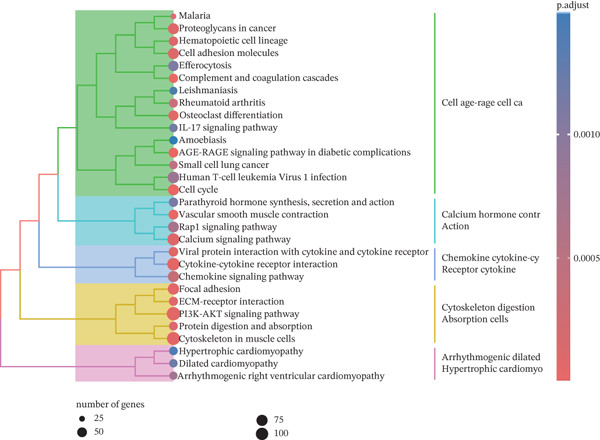
(f)

### 3.2. Results of Functional Enrichment Analysis

GO analysis showed that DEGs were significantly enriched in extracellular matrix (ECM) organization (Figure 2e). KEGG pathway analysis revealed that DEGs were significantly enriched in the cell cycle pathway (*p* < 0.05) (Figure 2f). These findings strongly suggest that DEGs play crucial roles in regulating cell proliferation and facilitating signal transduction processes.

### 3.3. WGCNA

WGCNA clustering showed no outlier samples (Figure 3a,b). When the soft threshold was set to 10, the network exhibited a scale‐free distribution (Figure 3c). Ultimately, 12 modules were identified (Figures 3d, 3e, 3f, 3g, and 3h; Supporting Information 1: Figure S1A–N), containing 154 genes in total. Protein–protein interaction (PPI) analysis was performed on these 154 genes (Supporting Information 1: Figure S1O).

Figure 3WGCNA. (a) Sample dendrogram based on average linkage clustering with a red line indicating the outlier filtering threshold (20,000). (b) Integrated sample clustering tree and clinical trait heatmap highlighting the distribution of control and treat groups. (c) Selection of the optimal soft‐thresholding power based on scale‐free topology fit (threshold = 0.8) and mean connectivity. (d) Gene dendrogram constructed using hierarchical clustering based on topological overlap matrix (TOM) dissimilarity. (e) Initial module identification via the dynamic tree cut method, represented by the color bar below the dendrogram. (f) Hierarchical clustering of module eigengenes (MEs) with a red line (0.25) indicating the threshold for merging highly correlated modules. (g) Final gene modules assigned after merging similar eigengenes to refine the coexpression network. (h) Module‐trait relationship heatmap displaying correlation coefficients and corresponding values (in parentheses) for each module and clinical trait.

**Figure:**
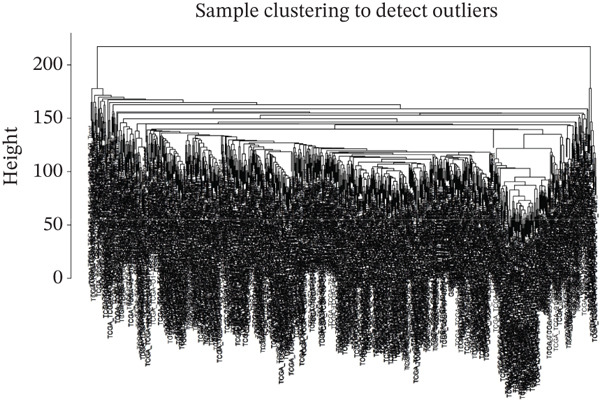
(a)

**Figure:**
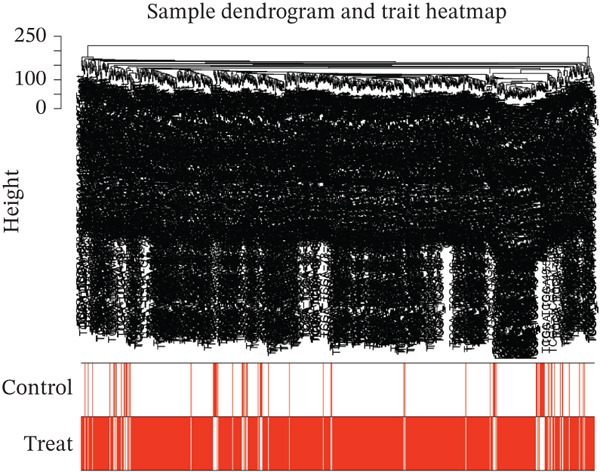
(b)

**Figure:**
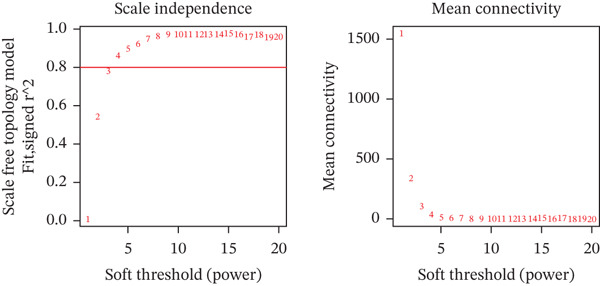
(c)

**Figure:**
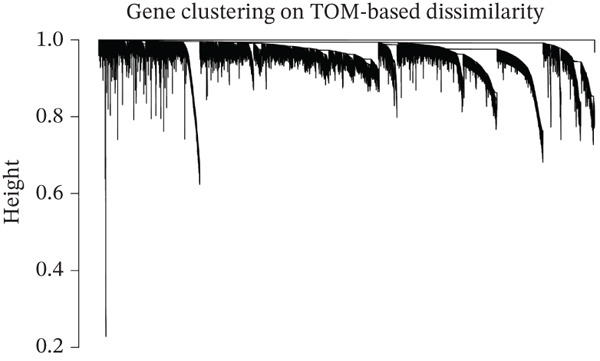
(d)

**Figure:**
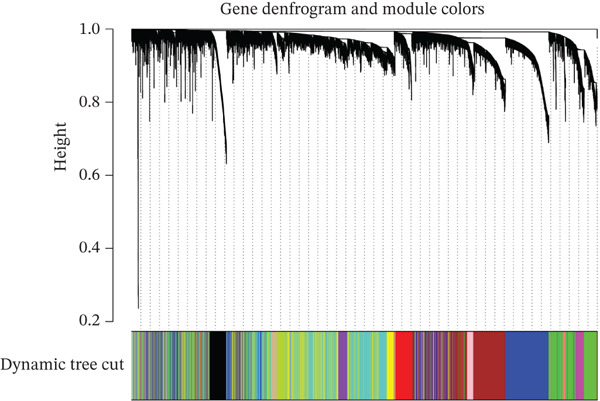
(e)

**Figure:**
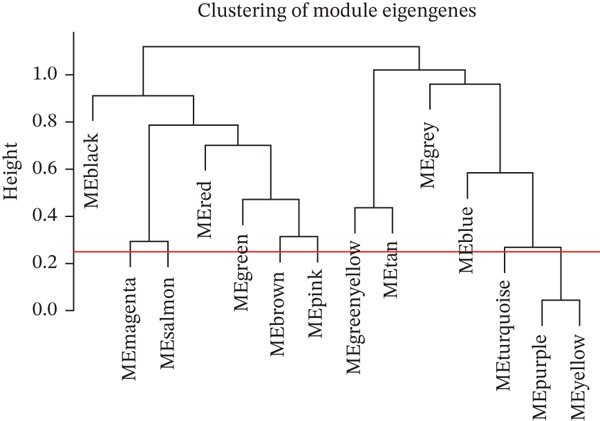
(f)

**Figure:**
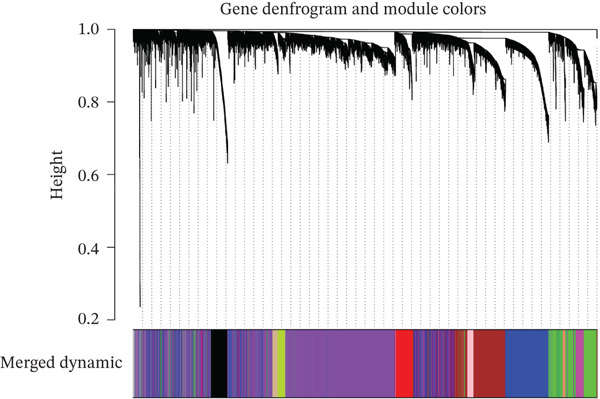
(g)

**Figure:**
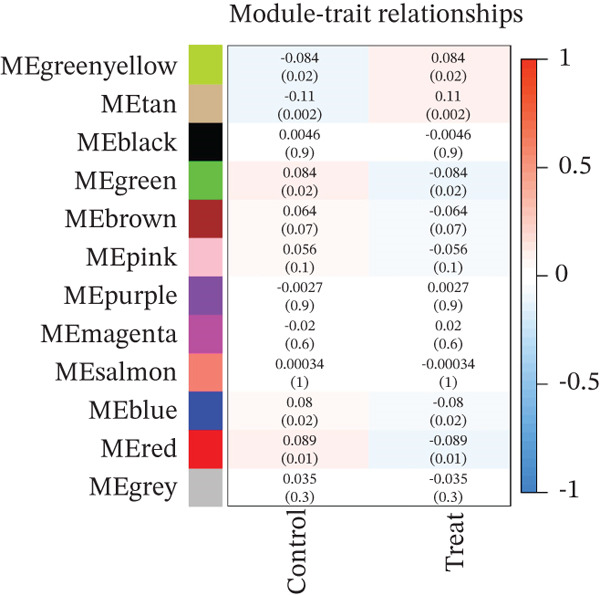
(h)

### 3.4. Feature Gene Selection and Model Performance

Three algorithms were used in this study: LASSO regression, SVM‐RFE, and RF. LASSO regression effectively screened seven core genes (Figure 4a,b); the SVM‐RFE algorithm further optimized feature selection and identified seven core genes (Figure 4c,d); in addition, the RF algorithm also screened seven core genes, with detailed results shown in Figure 4e,f. A total of seven common genes were identified: TTC13, TAF1D, ZNF587, PRPF3, long intergenic noncoding RNA 01355 (LINC01355), transactivation response RNA‐binding Protein 1 (TARBP1), and cyclin L2 (CCNL2). These seven genes were designated as “model genes.”

Figure 4Feature gene screening via machine learning. (a) LASSO coefficient trajectories of candidate genes across penalty parameter values. (b) Selection of the optimal penalty parameter (*λ*) via 10‐fold cross‐validation. (c) SVM‐RFE feature selection based on cross‐validation error rates. (d) SVM‐RFE classification accuracy across different gene numbers. (e) Random forest model convergence showing classification error versus tree number. (f) Variable importance ranking of the top genes based on Mean Decrease Gini values.

**Figure:**
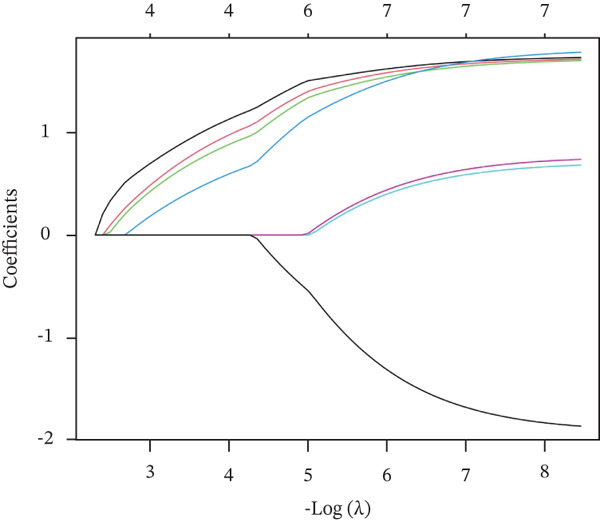
(a)

**Figure:**
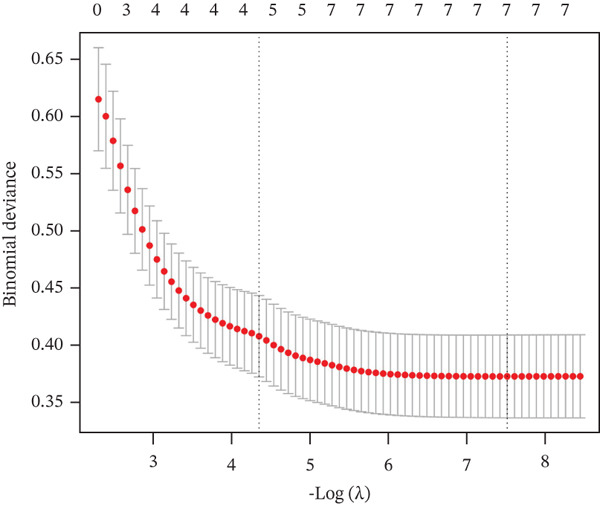
(b)

**Figure:**
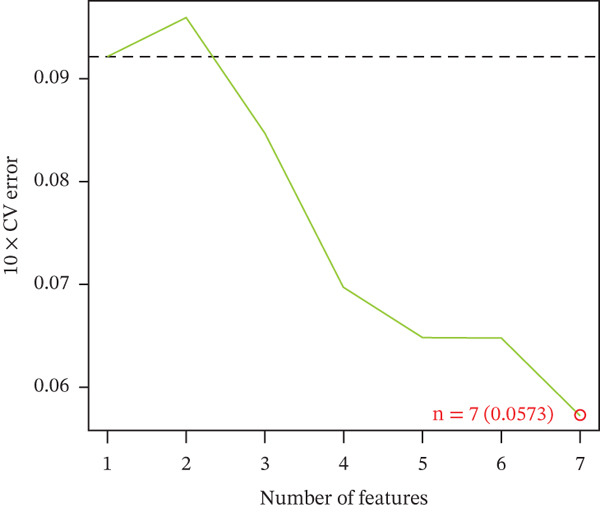
(c)

**Figure:**
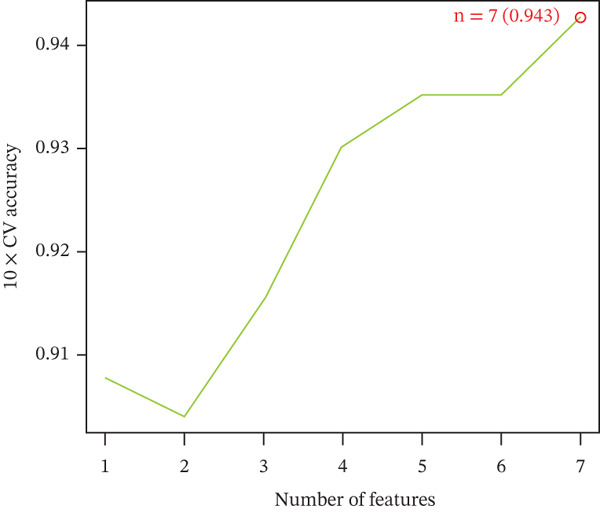
(d)

**Figure:**
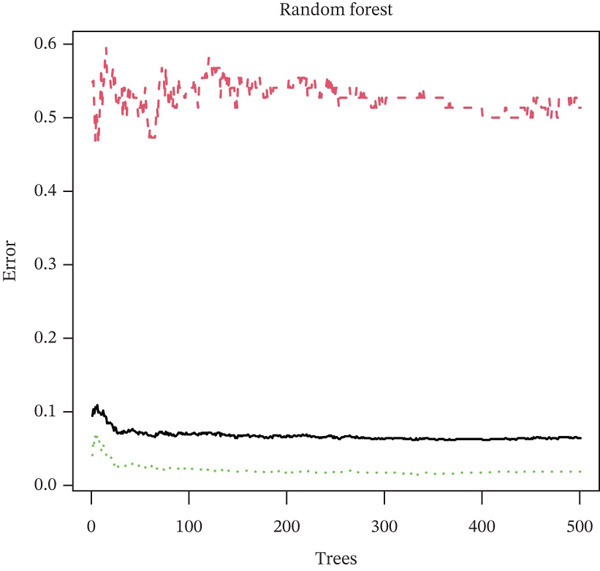
(e)

**Figure:**
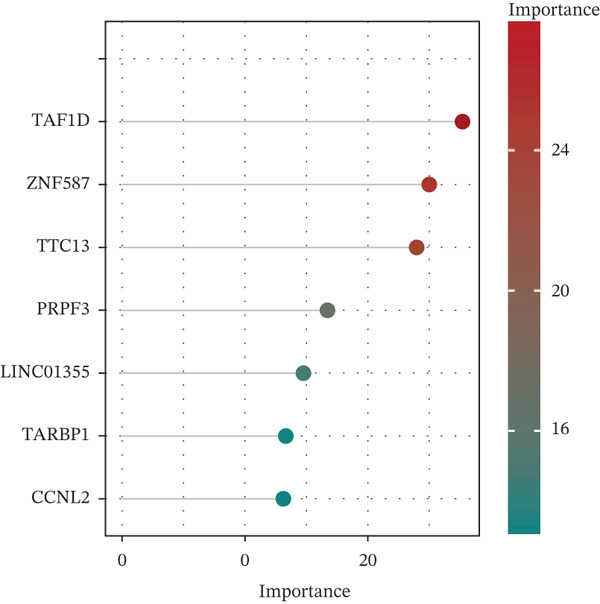
(f)

### 3.5. Contribution Analysis of Key Genes

SHAP analysis indicated that the seven core genes made significant contributions to classification (Figure 5a,b). Among them, TAF1D had the highest mean absolute SHA*P* value (0.0240), suggesting that changes in its expression had the greatest impact on prediction results. The dependence plot showed that TTC13, TAF1D, ZNF587, PRPF3, LINC01355, and TARBP1 exhibited a nonlinear positive correlation with SHAP values, while CCNL2 showed a nonlinear negative correlation with SHAP values (Figure 5c). A single‐sample force plot intuitively illustrated the cumulative effect of each gene on the prediction probability (Figure 5d). A waterfall plot revealed that the positive contributions of LINC01355 and TAF1D were the main drivers of the prediction results (Figure 5e).

Figure 5Model evaluation using the SHAP algorithm. (a) Global SHAP importance ranking of genes based on mean absolute SHA*P* values. (b) SHAP beeswarm plot showing the distribution and direction of gene‐specific effects across samples. (c) SHAP dependence plots illustrating the relationship between normalized gene expression and SHAP values. (d) SHAP force plot visualizing feature‐specific positive and negative influences on the classification outcome. (e) SHAP waterfall plot illustrating the additive decomposition of the model output for the first sample from the baseline (expected value) to the final prediction. (f) ROC curve comparison of machine learning models with AUC values indicating predictive performance.

**Figure:**
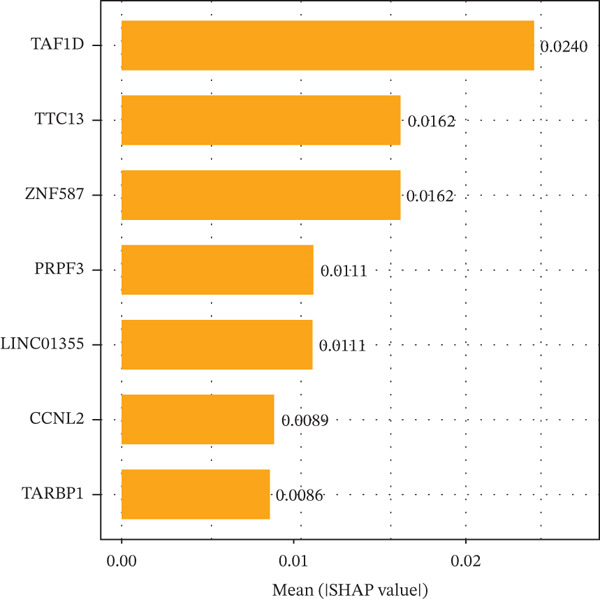
(a)

**Figure:**
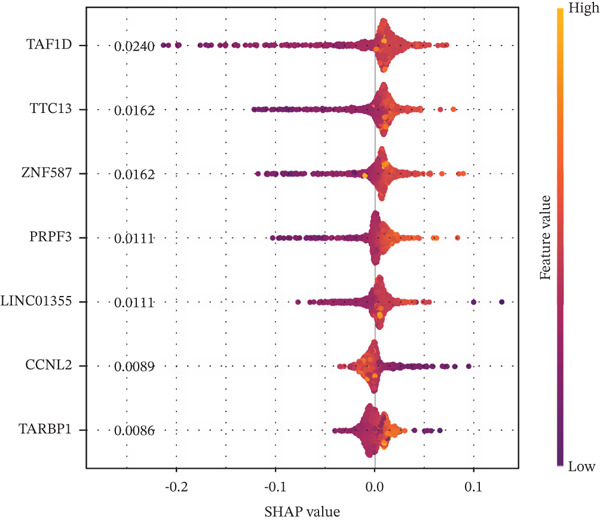
(b)

**Figure:**
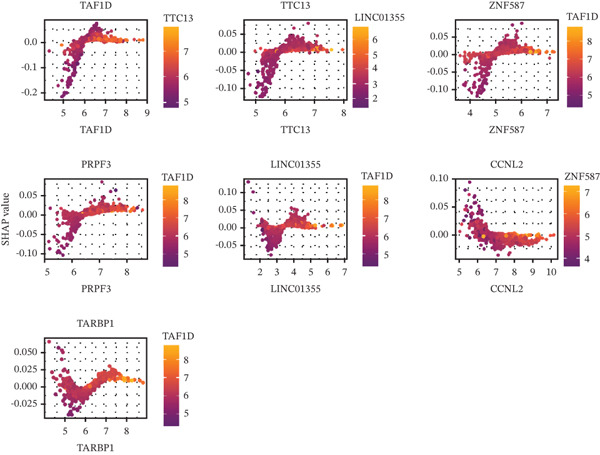
(c)

**Figure:**
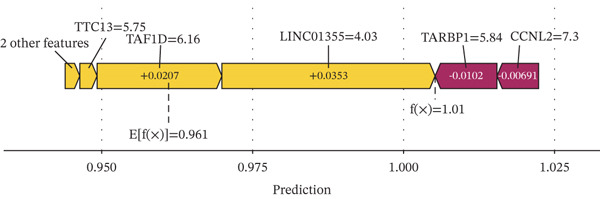
(d)

**Figure:**
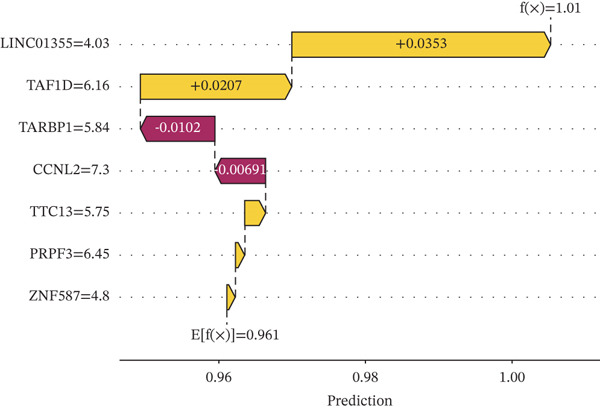
(e)

**Figure:**
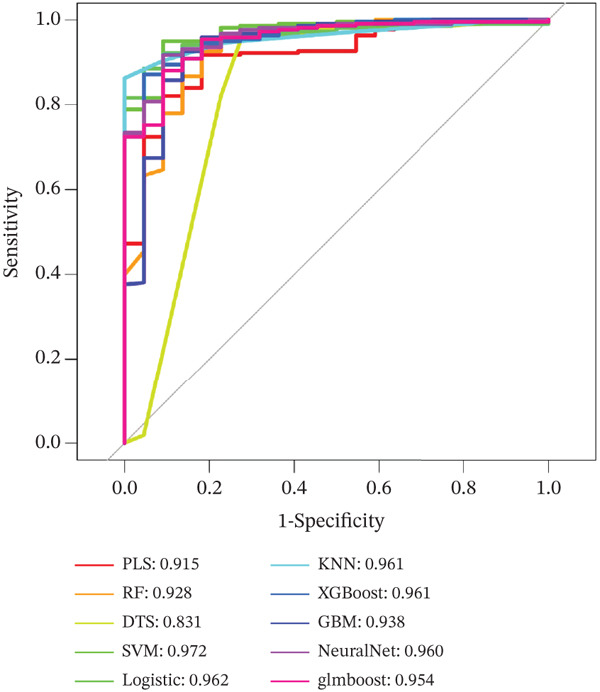
(f)

### 3.6. Comparison of Model Performance

As shown in Figure 5f, the AUC values of the 10 constructed models ranged from 0.831 to 0.972. Among these models, the support vector machine (SVM) exhibited the best performance, achieving an AUC value of 0.972; in contrast, the decision tree (DT) showed relatively poor efficacy, with an AUC value below 0.90.

### 3.7. Model Gene Correlation Analysis and Immune Analysis

Compared with the normal group, the expression levels of these seven genes were significantly upregulated in the tumor group (Figure 6a). The associations between the seven genes were statistically significant (Figure 6b), which was further confirmed by PPI analysis (Figure 6c). To further explore the biological significance of the model genes, GSEA analysis was conducted (Figure 6d). Given that molecular functions were closely associated with the immune microenvironment, the correlations between model genes and immune cells were analyzed. Bar chart analysis showed distinct differences in immune cell profiles between the two groups; most immune cell populations exhibited significant intercorrelations (*p* < 0.05) (Supporting Information 2: Figure S2A). Box‐and‐whisker plots demonstrated that the content of most immune cells was significantly higher in the tumor group than in the control group (*p* < 0.05) (Supporting Information 2: Figure S2B). Heatmap analysis revealed that TTC13, TAF1D, ZNF587, PRPF3, LINC01355, TARBP1, and CCNL2 were correlated with most immune cells (*p* < 0.05) (Figure 6e). The AUC values of TTC13, TAF1D, ZNF587, PRPF3, LINC01355, TARBP1, and CCNL2 were generally greater than 0.70 (Figure 6f).

Figure 6Model gene correlation analysis and immune analysis. (a) Box plots showing differential normalized expression of diagnostic genes between control (blue) and treat (red) groups (^***^p < 0.001). (b) Gene expression correlation matrix in the treat group displaying expression distributions, regression relationships, and Pearson correlation coefficients. (c) Protein–protein interaction (PPI) network illustrating predicted interactions among candidate diagnostic genes. (d) Functional enrichment analysis of significantly enriched biological processes or pathways associated with the identified gene signatures. (e) Integrated gene–immune cell association landscape presenting Spearman correlations and their direction and strength. (f) ROC curve analysis with AUC values evaluating the diagnostic performance of individual genes.

**Figure:**
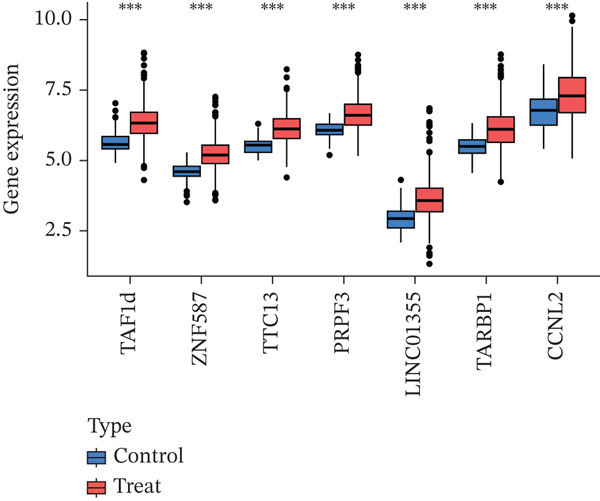
(a)

**Figure:**
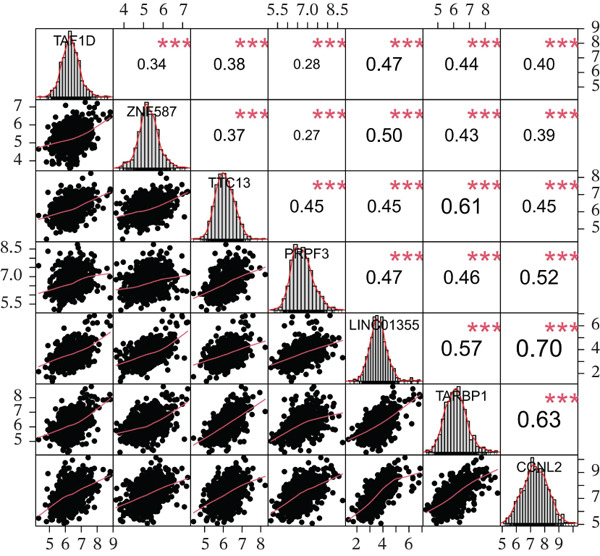
(b)

**Figure:**
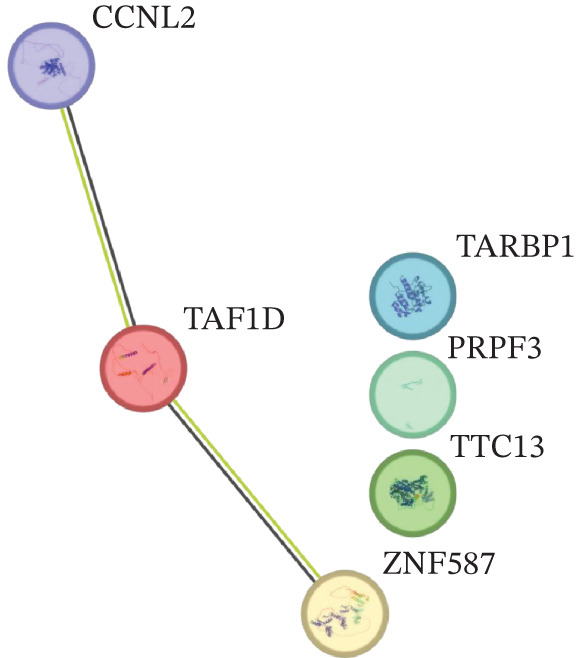
(c)

**Figure:**
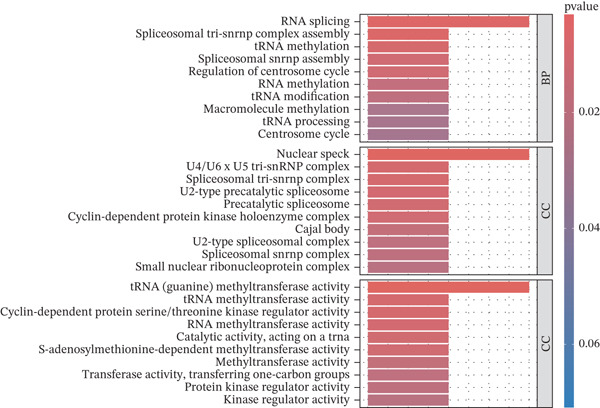
(d)

**Figure:**
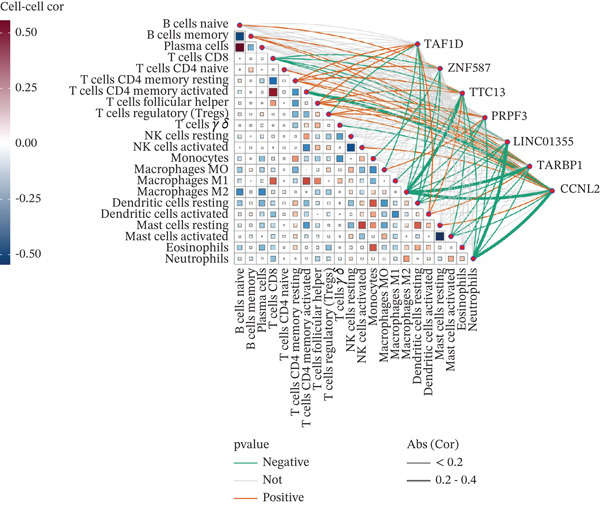
(e)

**Figure:**
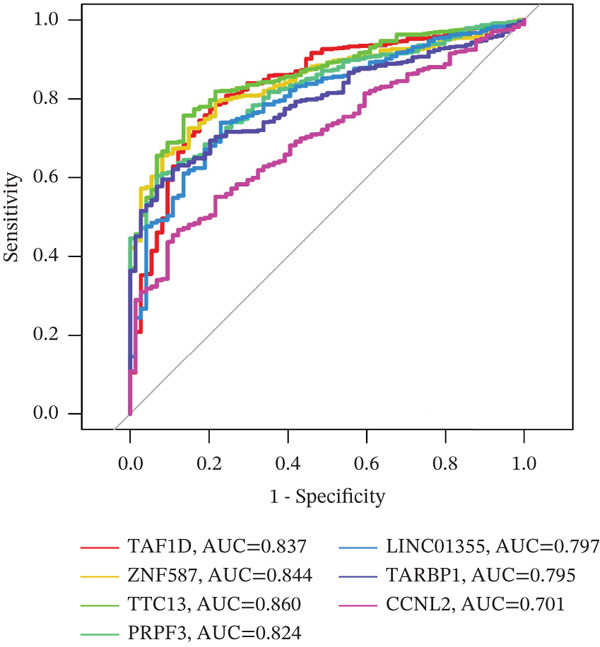
(f)

### 3.8. Single‐Cell Analysis

The single‐cell clustering results of normal lung tissue samples are shown in Figure 7a, and the cell annotations are presented in Figure 7b; subsequently, the expression of the seven model genes was visualized (Figure 7c,d). For LUAD samples, the single‐cell clustering results are displayed in Figure 7e, the cell annotations in Figure 7f, and the visualization of the seven model genes in Figure 7g,h.

Figure 7Single‐cell RNA‐seq analysis plots. (a) t‐SNE visualization of unsupervised clustering in the normal group. (b) Cell type annotation of normal cells identified by SingleR. (c) Dot plot showing lineage‐specific marker expression in the normal group. (d) Feature plots displaying canonical marker expression in the normal group. (e) t‐SNE visualization of unsupervised clustering in the tumor group. (f) Cell type annotation of tumor cells identified by SingleR. (g) Dot plot showing lineage‐specific marker expression in the tumor group. (h) Feature plots displaying canonical marker expression in the tumor group.

**Figure:**
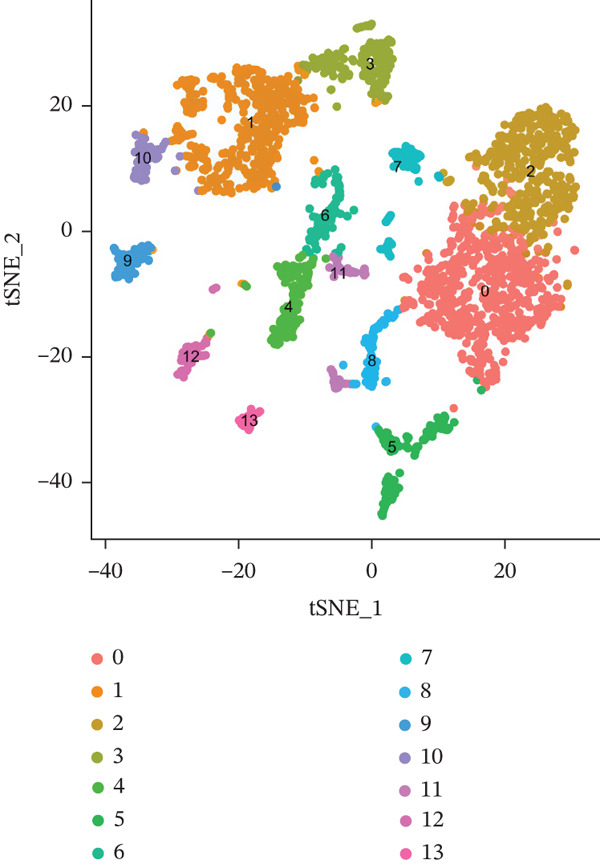
(a)

**Figure:**
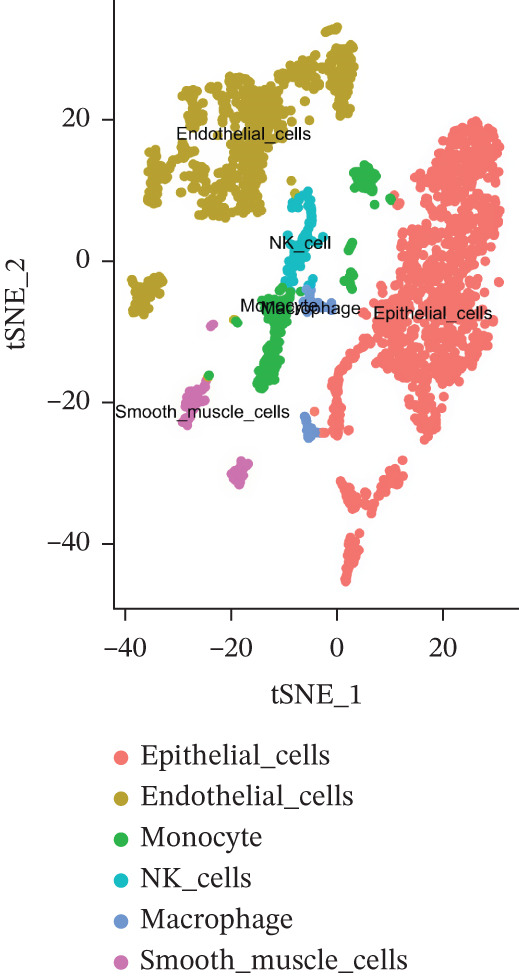
(b)

**Figure:**
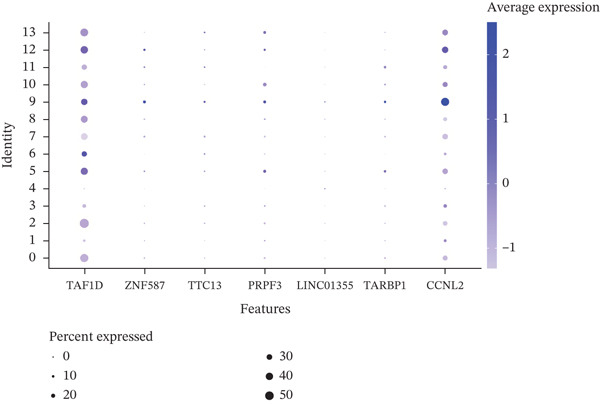
(c)

**Figure:**
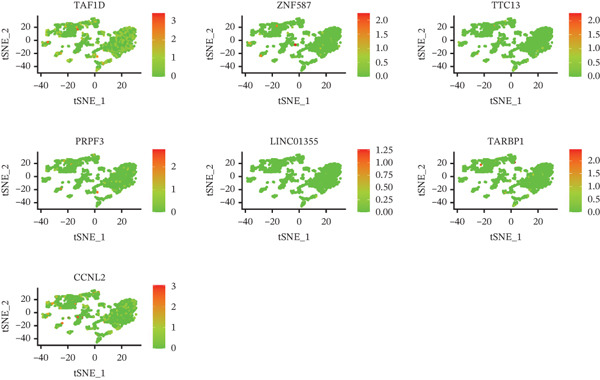
(d)

**Figure:**
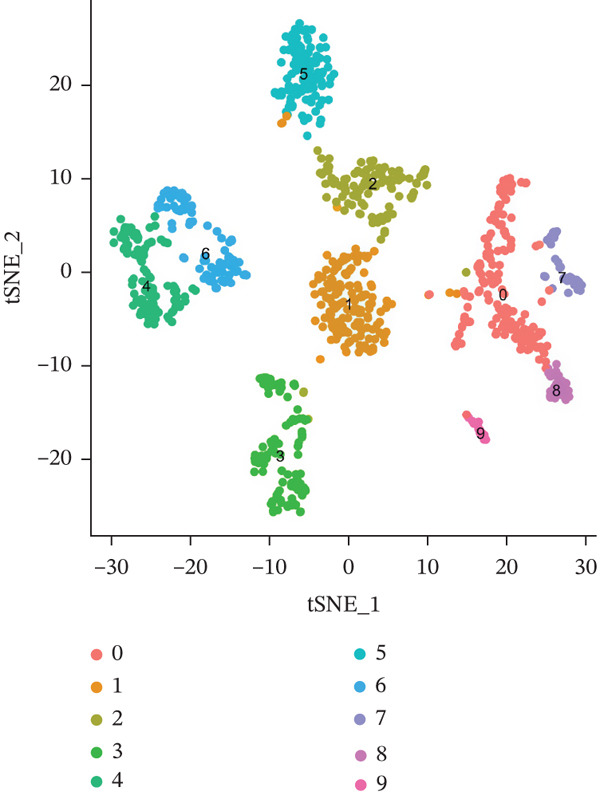
(e)

**Figure:**
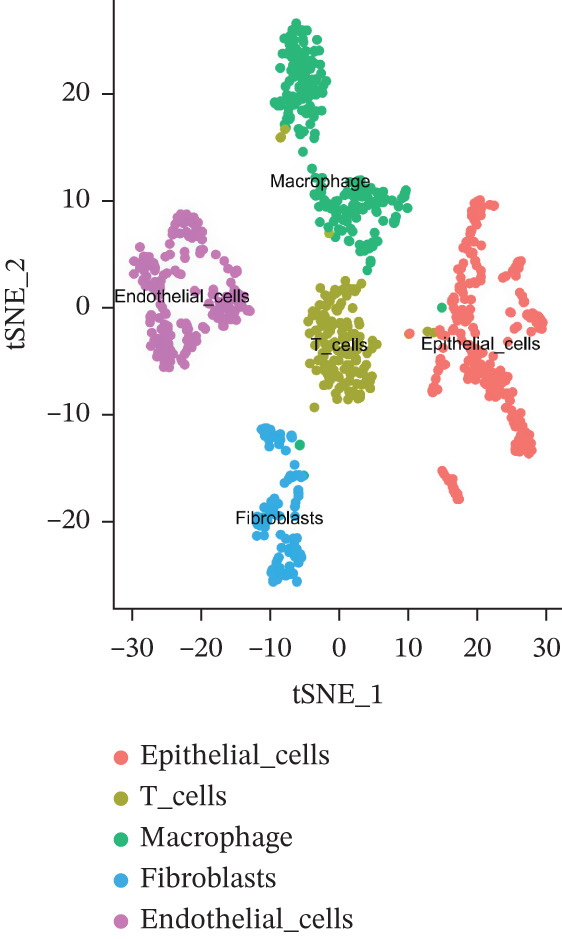
(f)

**Figure:**
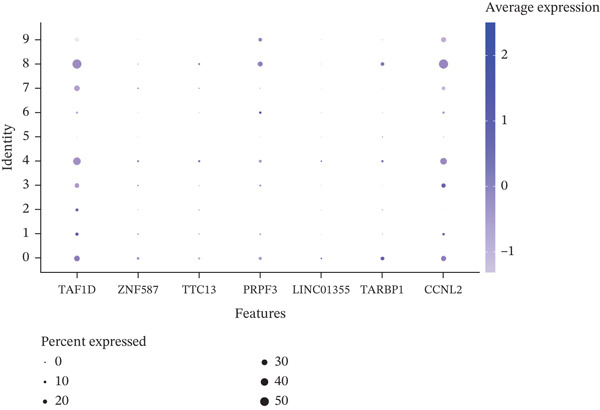
(g)

**Figure:**
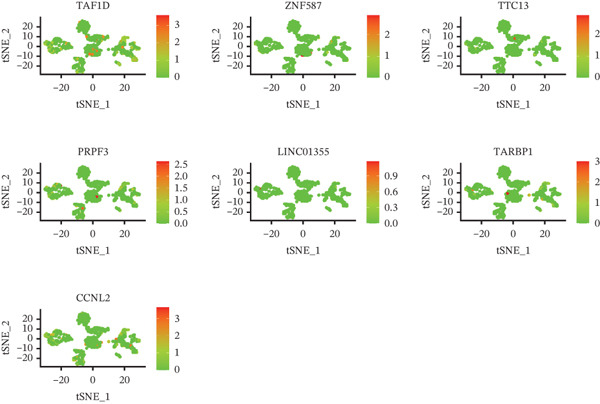
(h)

### 3.9. Clinically Relevant Analysis of TAF1D

TAF1D was widely expressed in healthy human tissues (Supporting Information 3: Figure S3A) and significantly upregulated in the tumor group (Supporting Information 3: Figure S3B). However, TAF1D expression showed no statistical significance across tumor stages, lymph node stages, ethnicities, genders, smoking statuses, or age groups (Supporting Information 3: Figure S3C–H), indicating that TAF1D is a potential biomarker and prognostic factor for LUAD (Supporting Information 3: Figure S3I). Meanwhile, TAF1D could also serve as a potential biomarker for other types of tumors (Supporting Information 3: Figure S3J).

### 3.10. Somatic Alteration Landscape of TAF1D in LUAD

Somatic mutation analysis of LUAD from the TCGA Pan‐Cancer Atlas cohort revealed that TAF1D exhibited a low overall alteration frequency of approximately 3% (Supporting Information 4: Figure S4A). Lollipop analysis demonstrated that TAF1D mutations were sparsely distributed across the protein‐coding region without evident hotspot clustering. Only a few isolated missense and frameshift mutations were observed, including a frameshift alteration (N143Dfs∗8) (Supporting Information 4: Figure S4B). Kaplan–Meier survival analysis showed no statistically significant difference in overall survival between patients with and without TAF1D alterations, suggesting that TAF1D somatic mutation alone may not serve as an independent prognostic factor in LUAD (Supporting Information 4: Figure S4C).

### 3.11. Comutation Analysis Reveals Enrichment in TP53‐Mutated Tumors

To further characterize the genomic context of TAF1D alterations, comutation analysis was performed. Consistent with established LUAD molecular patterns, EGFR and KRAS mutations displayed significant mutual exclusivity (log_2_OR = −1.771, *q* < 0.001), confirming dataset robustness. EGFR alterations significantly co‐occurred with TP53 mutations (log_2_OR = 1.256, *q* < 0.001), whereas KRAS mutations exhibited mutual exclusivity with TP53 (log_2_OR = −0.958, *q* = 0.002). Notably, TAF1D alterations demonstrated significant co‐occurrence with TP53 mutations (log_2_OR = 2.060, *q* = 0.029). This enrichment indicates that TAF1D genomic alterations preferentially arise in tumors harboring TP53 mutations. In contrast, no significant association was observed between TAF1D and EGFR or KRAS alterations. Collectively, these findings suggest that although TAF1D is infrequently altered in LUAD, its alterations are nonrandomly distributed and may be associated with a TP53‐mutated genomic background (Supporting Information 6: Table S1).

### 3.12. Spatial Heterogeneity of TAF1D Expression

Figure 8 clearly shows that TAF1D expression varied significantly among different samples (Figures 8a, 8b, 8c, 8d, 8e, 8f, 8g, 8h, 8i, 8j, 8k, 8l, 8m, 8n, and 8o). Different color patterns in tissue sections indicated that the expression of this gene differed across spatial regions, and genes in these regions might have distinct functions.

Figure 8(a–o) Spatial plots of the target gene (TAF1D).

**Figure:**
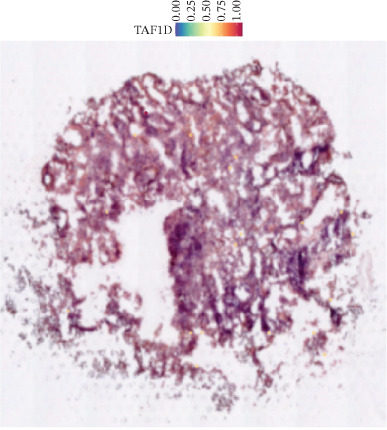
(a)

**Figure:**
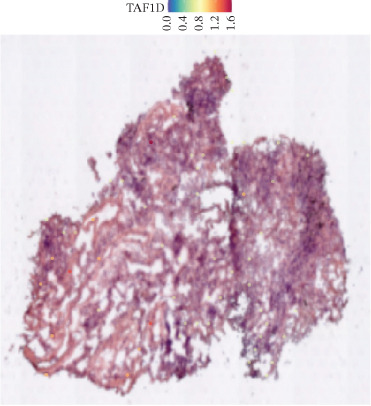
(b)

**Figure:**
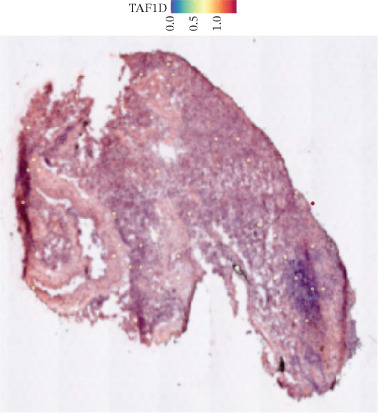
(c)

**Figure:**
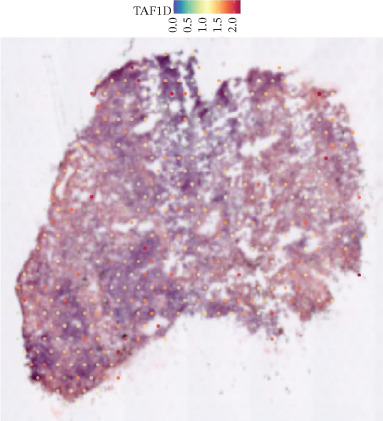
(d)

**Figure:**
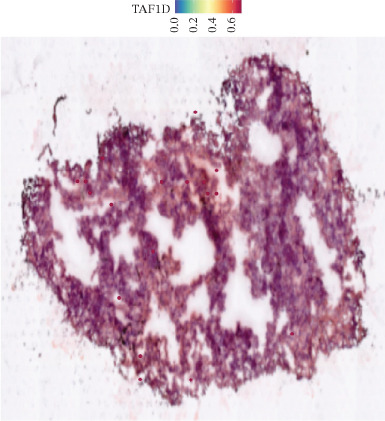
(e)

**Figure:**
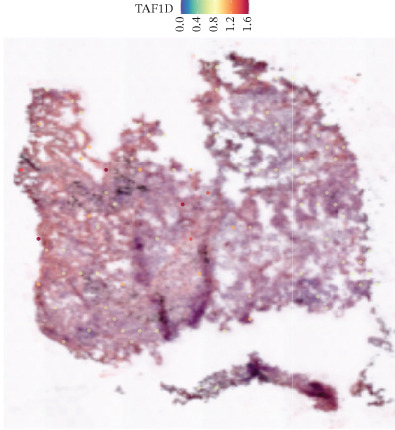
(f)

**Figure:**
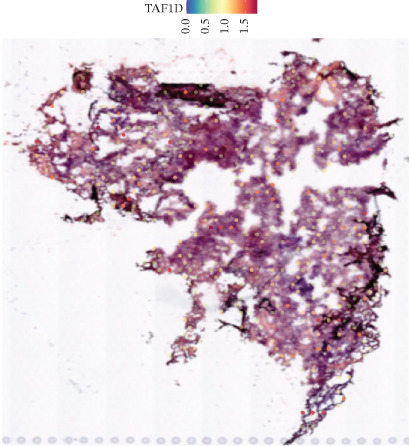
(g)

**Figure:**
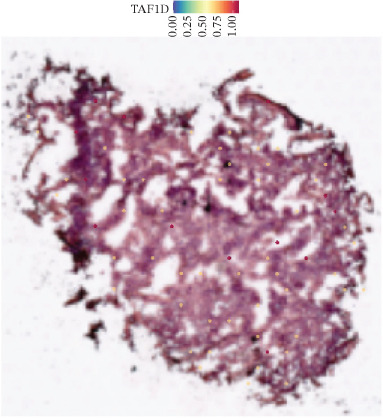
(h)

**Figure:**
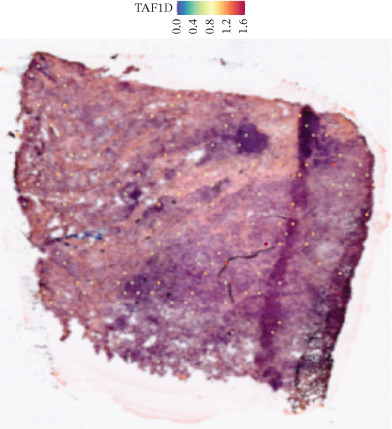
(i)

**Figure:**
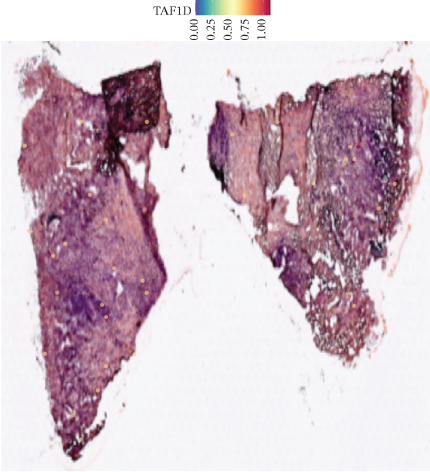
(j)

**Figure:**
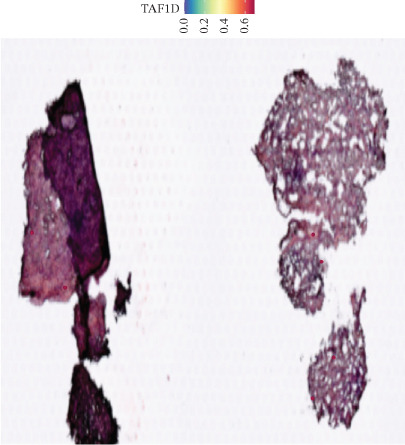
(k)

**Figure:**
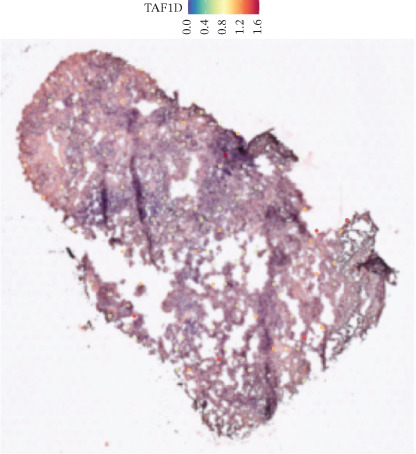
(l)

**Figure:**
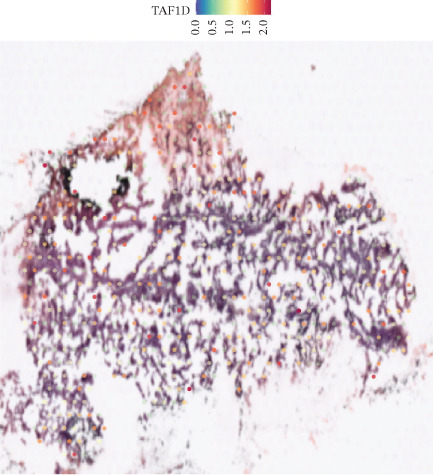
(m)

**Figure:**
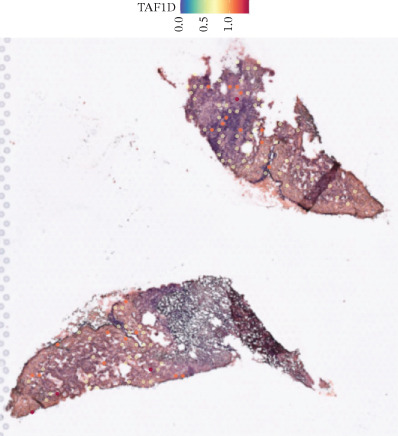
(n)

**Figure:**
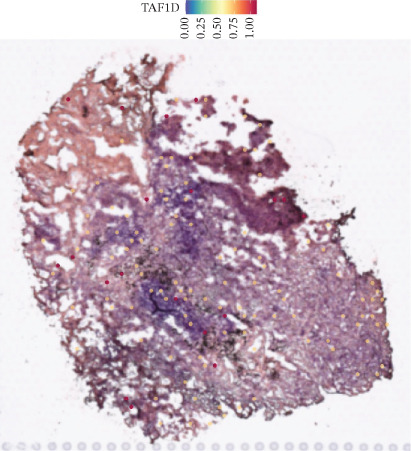
(o)

### 3.13. Downstream Research on TAF1D

GSEA analysis revealed that TAF1D could induce changes in downstream signaling pathways such as the cell cycle (Figure 9a,b) and simultaneously regulate molecular functions such as immune receptors (Figure 9c,d). The upstream and downstream regulators of TAF1D were also predicted using the GeneMANIA database (https://genemania.org/)(Figure 9e), and the downstream targets of TAF1D were predicted through gene knockout simulation (Figure 9f).

Figure 9Mechanism prediction of the target gene. (a) GSEA functional enrichment plot of the TAF1D high‐expression group. (b) GSEA functional enrichment plot of the TAF1D low‐expression group. (c) GSEA signaling pathway enrichment plot of the TAF1D high‐expression group. (d) GSEA signaling pathway enrichment plot of the TAF1D low‐expression group. (e) Upstream and downstream prediction of target gene TAF1D. (f) Expression changes of downstream molecules after TAF1D knockout in single‐cell samples.

**Figure:**
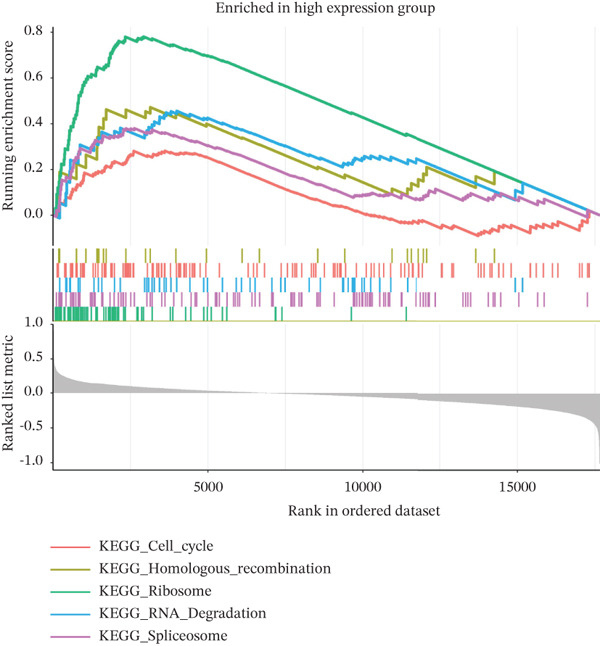
(a)

**Figure:**
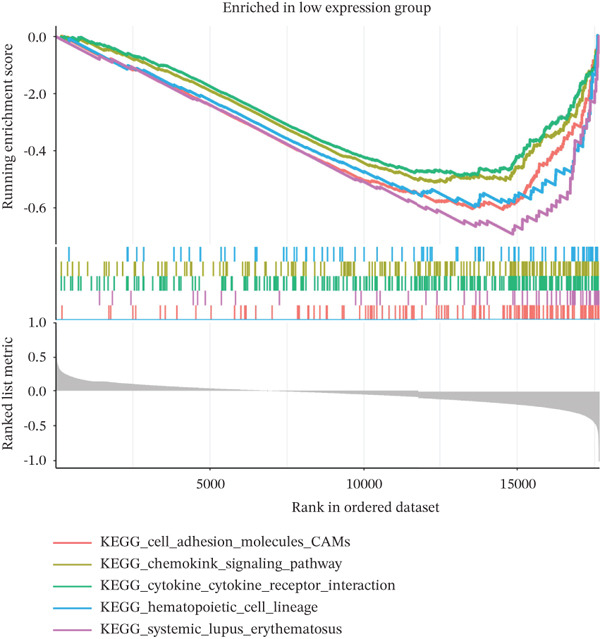
(b)

**Figure:**
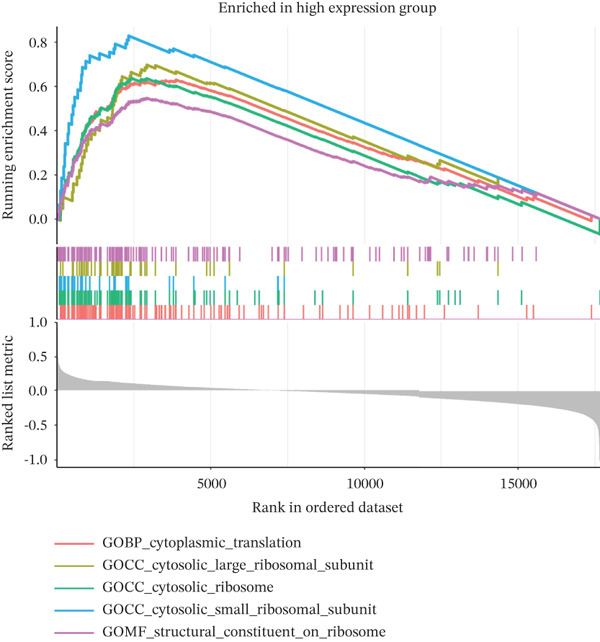
(c)

**Figure:**
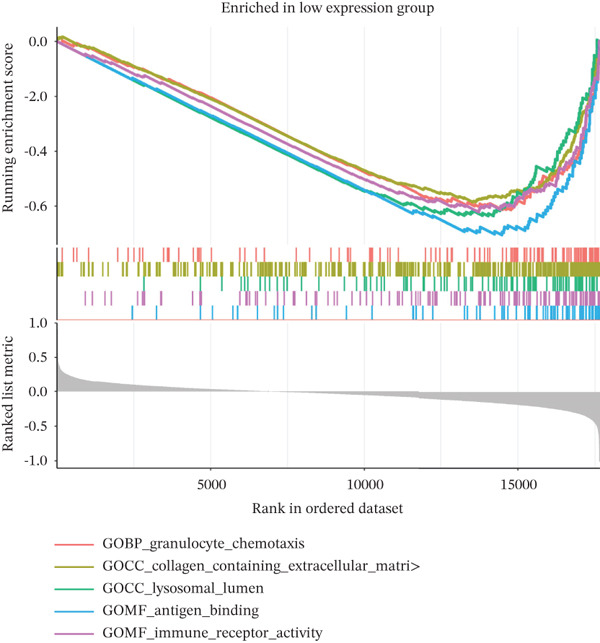
(d)

**Figure:**
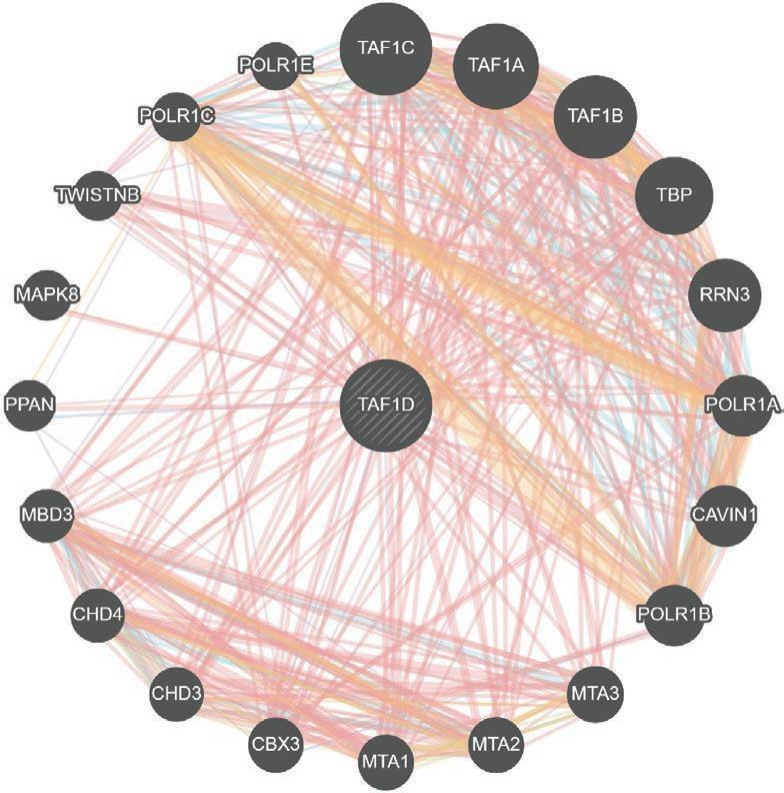
(e)

**Figure:**
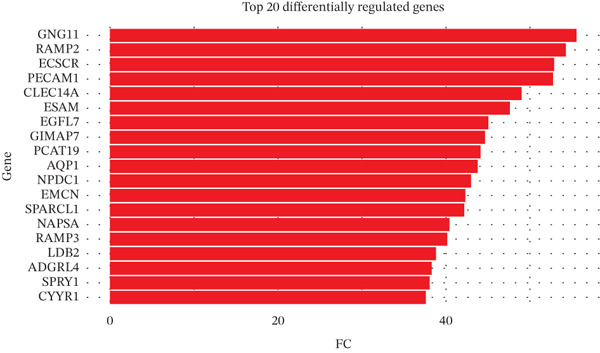
(f)

### 3.14. Exploration of TAF1D Function

Figure 10a shows the relative mRNA expression levels of TAF1D in AEC, A549, and PC‐9 cell lines, with significant differences observed among the cell lines. We selected the PC‐9 cell line as a model cell for functional experiments. Figure 10b shows the validation of TAF1D knockdown efficiency, and Figure 10c shows the validation of TAF1D overexpression efficiency (Supporting Information 5: Figure S5A). Figure 10d,e indicates that TAF1D can affect the proliferative function of LUAD cell lines.

Figure 10Model gene validation and functional experiment validation. (a) TAF1D expression levels in AEC, A549, and PC‐9 cells. (b) Validation of TAF1D knockdown efficiency in the PC‐9 cell line. (c) Validation of TAF1D overexpression efficiency in the PC‐9 cell line. (d) CCK‐8 assay in TAF1D knockdown cell model. (e) CCK‐8 assay in TAF1D overexpression cell model (^*^p<0.05).

**Figure:**
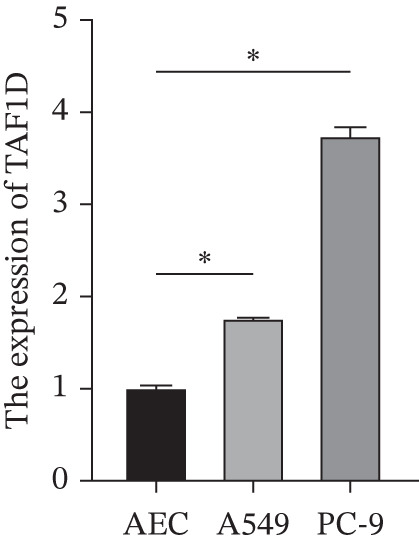
(a)

**Figure:**
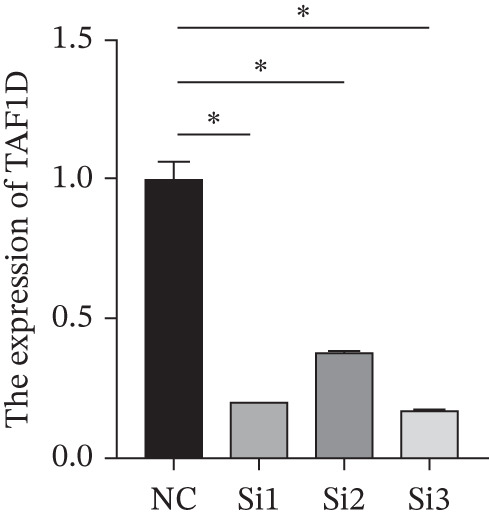
(b)

**Figure:**
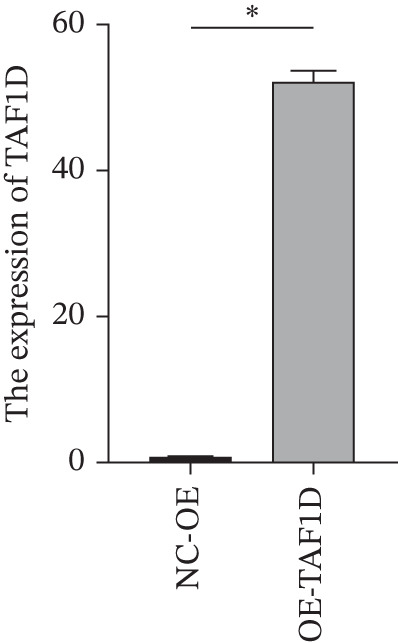
(c)

**Figure:**
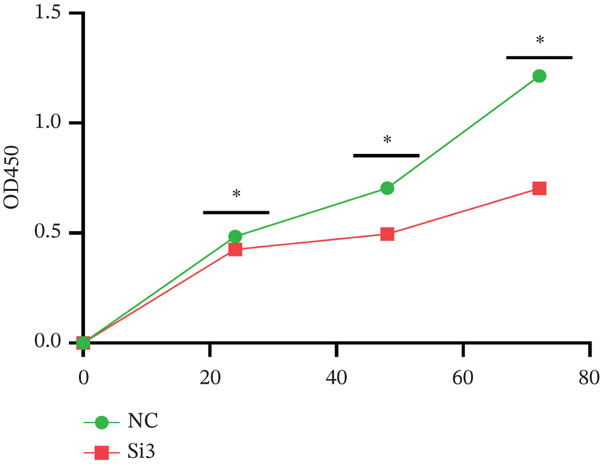
(d)

**Figure:**
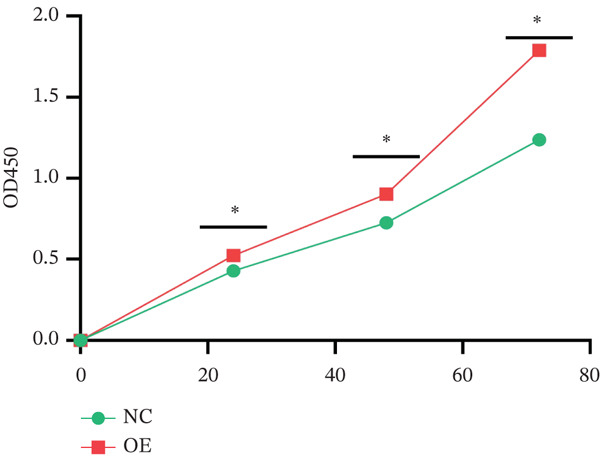
(e)

## 4. Discussion

This study systematically identified a panel of LUAD‐associated model genes (TTC13, TAF1D, ZNF587, PRPF3, LINC01355, TARBP1, and CCNL2) and delineated the clinical and functional significance of TAF1D through integrated multidataset analysis, machine learning, single‐cell profiling, and in vitro validation. The findings not only address critical gaps in current LUAD biomarker research but also advance our understanding of the molecular mechanisms underlying LUAD progression, with implications for improving diagnosis and therapeutic strategy development.

TAF1D has been confirmed to promote cell proliferation and induce G2/M phase arrest in MYCN‐amplified neuroblastoma by transcriptionally activating G2/M phase–related genes, including cyclin‐dependent Kinase 1 (CDK1) [23]. In clear cell renal cell carcinoma (ccRCC), high TAF1D expression is associated with poor patient survival; it promotes tumorigenesis and metastasis by activating the PI3K/AKT/mTOR signaling pathway [24]. Additionally, high TAF1D expression has been found to correlate with adverse prognosis in both ccRCC and osteosarcoma, making it a potential novel biomarker [24, 25].

The ZNF587 gene, full name Zinc Finger Protein 587, belongs to the family of KRAB‐containing zinc finger proteins (KZFPs). KZFPs are important epigenetic regulators that modulate gene expression by influencing heterochromatin at regulatory sequences embedded with transposable elements (TEs), playing a key role in maintaining genomic stability and cellular functions [26]. A preprint study published on bioRxiv indicated that the KZFPs ZNF587/ZNF417 are associated with adverse prognosis in diffuse large B‐cell lymphoma (DLBCL) [26].

LINC01355 is a long noncoding RNA (lncRNA). LncRNAs play crucial roles in regulating gene expression and exhibit abnormal expression and function in various cancers. Studies have shown that LINC01355 is downregulated in breast cancer cells; overexpression of LINC01355 can significantly inhibit breast cancer cell growth and induce cell cycle arrest and apoptosis. The underlying mechanism may involve FOXO3‐mediated transcriptional repression of CCND1 [23].

TARBP1 is an RNA‐binding protein involved in RNA processing and translational regulation. Research has explored the expression of TARBP1 protein in NSCLC and its prognostic significance [25]. In this study, RT‐qPCR was used to detect TARBP1 mRNA expression in 10 pairs of NSCLC tissues and matched adjacent normal tissues, while immunohistochemistry (IHC) was employed to analyze 90 paraffin‐embedded NSCLC tissue samples. The results demonstrated that TARBP1 expression levels in NSCLC and their association with clinicopathological features have prognostic significance [25]. Furthermore, the expression and prognostic value of TARBP1 in hepatocellular carcinoma (HCC) have also been analyzed, suggesting that it may play a role in multiple cancer types [27]. Therefore, in LUAD, TARBP1 may regulate the expression of oncogenes or tumor suppressor genes by affecting RNA stability, translational efficiency, or the function of noncoding RNAs, thereby influencing the occurrence and progression of LUAD.

CCNL2 is a cyclin involved in cell cycle regulation. A dysregulated cell cycle is one of the hallmark characteristics of tumors. A study found that the lncRNA lnc‐RNU12 affects T cell cycle progression through c‐JUN and CCNL2, thereby playing a role in rheumatoid arthritis (RA) [28]. While this study focused on RA, T cell cycle regulation is closely associated with immune responses and the tumor immune microenvironment. The immune microenvironment of LUAD is critical for tumor progression and treatment response [29]. Therefore, if CCNL2 is abnormally expressed in LUAD cells or immune cells (e.g., T cells) within the tumor immune microenvironment, it may promote tumor cell proliferation or modulate antitumor immune responses by influencing cell cycle progression. Further studies are needed to investigate the expression pattern of CCNL2 in LUAD, its impact on tumor cell proliferation and apoptosis, and its interaction with the tumor immune microenvironment.

TTC13 belongs to the tetratricopeptide repeat (TPR) protein family [30]. Members of this family are well‐known for their TPR domains, which primarily mediate PPIs and/or act as protein scaffolds [23, 30]. TPR proteins play key roles in various cellular processes, including protein folding, signal transduction, cell cycle regulation, and vesicular trafficking [30]. TTC13 may bind to other key signaling molecules or tumor‐associated proteins via its TPR domain, thereby regulating their activity, localization, or stability and further influencing tumor cell proliferation, apoptosis, or differentiation. For example, the TP53 tumor suppressor gene is mutated in approximately half of all human malignancies, and its function is controlled through posttranslational modifications and cofactor interactions [24, 31]. TRIP13 (thyroid hormone receptor–interacting Protein 13), an AAA+ ATPase, plays roles in DNA double‐strand break repair, chromosome synapsis, and cell cycle checkpoint regulation. Abnormal TRIP13 expression leads to chromosomal instability and is closely associated with adverse prognosis and tumor staging in various cancers [32, 33]. TRIP13 inhibits the proliferation and metastasis of thyroid cancer cells by regulating the TTC5/p53 pathway and the expression of epithelial–mesenchymal transition (EMT)–related genes [33]. While TTC13 differs from TTC5 or TRIP13, these examples highlight the importance of TPR family members in tumor suppressor pathways.

Although somatic mutation analysis revealed that TAF1D alterations occurred at a low frequency (~3%) and lacked recurrent hotspot mutations, comutation analysis demonstrated significant enrichment in TP53‐mutated tumors. This suggests that TAF1D alterations may arise within a TP53‐associated genomic instability background rather than functioning as classical driver mutations. Notably, despite its low mutation rate, TAF1D expression contributed to the prognostic model, indicating that its clinical relevance may be primarily mediated through transcriptional dysregulation rather than recurrent genomic mutations.

This study has several limitations. First, the initial DEG and model gene screening relied on public datasets, which may suffer from sample selection bias (e.g., underrepresentation of rare LUAD subtypes). Future studies should validate these findings in large, prospectively collected clinical cohorts. Second, functional experiments were limited to cell lines; animal models (e.g., xenografts) are needed to confirm TAF1D′s role in LUAD progression in vivo. Third, while we identified TAF1D′s downstream pathways (e.g., cell cycle), the specific molecular mechanisms (e.g., direct targets of TAF1D) remain unclear. ChIP‐seq or coimmunoprecipitation studies could help elucidate these mechanisms. Finally, the predictive model′s clinical utility (e.g., its ability to distinguish LUAD from benign lung diseases) was not tested; future work should compare its performance to existing diagnostic tools (e.g., CT or liquid biopsies).

Future directions could also explore the potential of our model genes as therapeutic targets. For example, TAF1D′s role in cell proliferation suggests that it could be targeted to inhibit tumor growth, while its correlation with immune cells opens avenues for combination immunotherapies. Additionally, the model genes′ collective predictive power could be integrated into a diagnostic panel, potentially improving early LUAD detection—especially in nonsmokers, who are often overlooked by traditional screening methods.

## 5. Conclusion

In summary, this study identifies a panel of seven LUAD model genes and characterizes TAF1D as a key biomarker with prognostic value and functional roles in cell proliferation and immune regulation. The SVM model, enhanced by SHAP analysis, provides an accurate and interpretable tool for LUAD diagnosis. These findings advance our understanding of LUAD biology and lay the groundwork for developing novel diagnostic and therapeutic strategies to improve patient outcomes.

## Author Contributions

Conceived and designed the experiments: Haitao Wang and Yanyan Shi; analyzed the data: Dongdong Liu and Jingyu Wu; wrote and revised the paper: Lan Ding and Qingmei Xu; drew the figures: Feiqi Xu, Xufan Cai, and Shuhan Ma. Lan Ding and Qingmei Xu contributed equally to this work.

## Funding

This work was funded by the Medicine and Health Research Foundation of Zhejiang Province (2022RC006 and 2025KY591).

## Ethics Statement

The authors have nothing to report.

## Consent

The authors have nothing to report.

## Conflicts of Interest

The authors declare no conflicts of interest.

## Supporting Information

Additional supporting information can be found online in the Supporting Information section.

## Supporting information


**Supporting Information 1** Figure S1: (A) Distribution of mean gene significance (GS) across modules. (B–N) Correlation between module membership (MM) and GS. (O) Protein–protein interaction (PPI) network.

**Supporting Information 2** Figure S2: (A) Landscape of immune cell infiltration. (B) Synergistic correlation of immune cells.

**Supporting Information 3** Figure S3: Clinical relevance analysis of TAF1D. (A) TAF1D expression levels in healthy human tissues. (B) TAF1D expression levels in the TCGA cohort. (C) TAF1D expression across different tumor stages. (D) TAF1D expression across different ethnicities. (E) TAF1D expression across different genders. (F) TAF1D expression across different age groups. (G) TAF1D expression across different smoking statuses. (H) TAF1D expression across different lymph node stages. (I) Prognostic impact of TAF1D high‐ and low‐expression groups. (J) TAF1D expression across 33 types of tumors.

**Supporting Information 4** Figure S4: Genomic landscape and comutation pattern of TAF1D in lung adenocarcinoma. (A) OncoPrint illustrating the alteration frequency and mutation types of TAF1D in the TCGA‐LUAD cohort. (B) Lollipop plot showing the distribution of TAF1D somatic mutations across the protein sequence. (C) Kaplan–Meier overall survival analysis comparing patients with and without TAF1D alterations.

**Supporting Information 5** Figure S5: (A) Western blot images of overexpression of TAF1D (right panel) and downregulation efficiency (left panel).

**Supporting Information 6** Table S1: Comutation and mutual exclusivity analysis of TAF1D and key driver genes in LUAD.

## Data Availability

The data that support the findings of this study are available from the corresponding authors upon reasonable request.

## References

[1] Succony L. , Rassl D. M. , Barker A. P. , McCaughan F. , and Rintoul R. C. , Adenocarcinoma Spectrum Lesions of the Lung: Detection, Pathology and Treatment Strategies, Cancer Treatment Reviews. (2021) 99, 102237, 10.1016/j.ctrv.2021.102237, 34182217. 34182217

[2] Shao D. , Su F. , Zou X. , Lu J. , Wu S. , Tian R. , Ran D. , Guo Z. , and Jin D. , Pixel-Level Classification of Five Histologic Patterns of Lung Adenocarcinoma, Analytical Chemistry. (2023) 95, no. 5, 2664–2670, 10.1021/acs.analchem.2c03020, 36701546. 36701546

[3] Jiang H. and Bu L. , Progress in the Treatment of Lung Adenocarcinoma by Integrated Traditional Chinese and Western Medicine, Frontiers in Medicine. (2024) 10, 10.3389/fmed.2023.1323344. PMC10802683 38259856

[4] Keerthana Priya P. , Ravichandar S. , Sampath S. , Verma G. , and Abraham E. A. , Myriad Presentation of Adenocarcinoma Lung, Journal of Evolution of Medical and Dental Sciences. (2024) 13, no. 2, 55–57, 10.14260/jemds.v13i2.558.

[5] Tang Q. , Li W. , Zheng X. , Ren L. , Liu J. , Li S. , Wang J. , and du G. , MELK Is an Oncogenic Kinase Essential for Metastasis, Mitotic Progression, and Programmed Death in Lung carcinoma, Signal Transduction and Targeted Therapy. (2020) 5, no. 1, 10.1038/s41392-020-00288-3. PMC7708490 33262323

[6] Coudray N. , Ocampo P. S. , Sakellaropoulos T. , Narula N. , Snuderl M. , Fenyö D. , Moreira A. L. , Razavian N. , and Tsirigos A. , Classification and mutation prediction from non–small cell lung cancer histopathology images using deep learning, Nature Medicine. (2018) 24, no. 10, 1559–1567, 10.1038/s41591-018-0177-5, 2-s2.0-85053661755, 30224757. PMC9847512 30224757

[7] Ding D. , Wang L. , Zhang Y. , Shi K. , and Shen Y. , Machine Learning Developed a Programmed Cell Death Signature for Predicting Prognosis and Immunotherapy Benefits in Lung Adenocarcinoma, Translational Oncology. (2023) 38, 101784, 10.1016/j.tranon.2023.101784, 37722290. 37722290 PMC10511492

[8] Hakkoum H. , Idri A. , and Abnane I. , Global and Local Interpretability Techniques of Supervised Machine Learning Black Box Models for Numerical Medical Data, Engineering Applications of Artificial Intelligence. (2024) 131, 107829, 10.1016/j.engappai.2023.107829.

[9] Lundberg S. and Lee S. I. , A Unified Approach to Interpreting Model Predictions, Proceedings of the 31st International Conference on Neural Information Processing Systems, 2017, ACM, 4768–4777.

[10] Giacobbe D. R. , Marelli C. , Guastavino S. , Mora S. , Rosso N. , Signori A. , Campi C. , Giacomini M. , and Bassetti M. , Explainable and Interpretable Machine Learning for Antimicrobial Stewardship: Opportunities and Challenges, Clinical Therapeutics. (2024) 46, no. 6, 474–480, 10.1016/j.clinthera.2024.02.010, 38519371. 38519371

[11] Petrovska N. , Larrondo-Petrie M. , and Pavlovic M. , Improving Model Explainability in AD Prediction: SHAP-Based Feature Attribution and Interpretable Ensembles, 2025 International Conference on Advanced Machine Learning and Data Science (AMLDS), 2025, IEEE, 175–180, 10.1109/AMLDS63918.2025.11159451.

[12] Sawant T. , A Comparative Study of SHAP and LIME: Interpreting Black-Box Machine Learning Models, INTERNATIONAL JOURNAL OF SCIENTIFIC RESEARCH IN ENGINEERING AND MANAGEMENT. (2025) 9, no. 7, 1–9, 10.55041/IJSREM51586.

[13] Singh K. , Kashyap A. , and Cherukuri A. K. , Interpretable Anomaly Detection in Encrypted Traffic Using SHAP With Machine Learning Models, 2025, https://arxiv.org/abs/2505.16261.

[14] Yang W. , Li H. , Wang J. , and Ma H. , Spatio-Temporal Feature Interpretable Model for Air Quality Forecasting, Ecological Indicators. (2024) 167, 112609, 10.1016/j.ecolind.2024.112609.

[15] Meiseles A. and Rokach L. , Iterative Feature eXclusion (IFX): Mitigating Feature Starvation in Gradient Boosted Decision Trees, Knowledge-Based Systems. (2024) 289, 111546, 10.1016/j.knosys.2024.111546.

[16] Rao Y. , Zhang L. , Gao L. , Wang S. , and Yang L. , ExAutoGP: Enhancing Genomic Prediction Stability and Interpretability With Automated Machine Learning and SHAP, Animals. (2025) 15, no. 8, 10.3390/ani15081172, 40282006. PMC12024354 40282006

[17] Happila T. , Simbu M. , Rajendran A. , Ranjith Kumar P. , Rajakumar S. , and Hariprakash P. , Enhancing Brain Tumor Diagnosis: A Hybrid Machine Learning Model With PCA and SHAP for Interpretability, Proceedings of the 2025 International Conference on Data Science, Agents & Artificial Intelligence (ICDSAAI), 2025, IEEE.

[18] Noonpakdee W. and Danrangab S. , Predicting Dew Point Temperatures: A Machine Learning Approach With SHAP Explanations, ASEAN Journal of Scientific and Technological Reports. (2025) 28, no. 2, 10.55164/ajstr.v28i2.255728.

[19] Sullivan R. S. and Longo L. , Explaining Deep Q-Learning Experience Replay With SHapley Additive exPlanations, Machine Learning and Knowledge Extraction. (2023) 5, no. 4, 1433–1455, 10.3390/make5040072.

[20] Ladbury C. , Li R. , Danesharasteh A. , Ertem Z. , Tam A. , Liu J. , Hao C. , Li R. , McGee H. , Sampath S. , Williams T. , Glaser S. , Khasawneh M. , Liao Z. , Lee P. , Ryckman J. , Shaikh P. , and Amini A. , Explainable Artificial Intelligence to Identify Dosimetric Predictors of Toxicity in Patients With Locally Advanced Non-Small Cell Lung Cancer: A Secondary Analysis of RTOG 0617, International Journal of Radiation Oncology • Biology • Physics. (2023) 117, no. 5, 1287–1296, 10.1016/j.ijrobp.2023.06.019, 37406826. 37406826

[21] Nfissi A. , Bouachir W. , Bouguila N. , and Mishara B. , Unveiling Hidden Factors: Explainable AI for Feature Boosting in Speech Emotion Recognition, Applied Intelligence. (2024) 54, no. 11-12, 7046–7069, 10.1007/s10489-024-05536-5.

[22] Omer B. , Jaf D. K. I. , Abdalla A. , Mohammed A. S. , Abdulrahman P. I. , and Kurda R. , Advanced Modeling for Predicting Compressive Strength in Fly Ash-Modified Recycled Aggregate Concrete: XGBoost, MEP, MARS, and ANN approaches, Innovative Infrastructure Solutions. (2024) 9, no. 3, 10.1007/s41062-024-01365-0.

[23] Ai B. , Kong X. , Wang X. , Zhang K. , Yang X. , Zhai J. , Gao R. , Qi Y. , Wang J. , Wang Z. , and Fang Y. , LINC01355 Suppresses Breast Cancer Growth Through FOXO3-Mediated Transcriptional Repression of CCND1, Cell Death &amp; Disease. (2019) 10, no. 7, 10.1038/s41419-019-1741-8, 2-s2.0-85068012319. PMC6594972 31243265

[24] Hu X. , Chen L. , Liu T. , Wan Z. , Yu H. , Tang F. , Shi J. , Chen Z. , Wang X. , and Yang Z. , TAF1D Promotes Tumorigenesis and Metastasis by Activating PI3K/AKT/mTOR Signaling in Clear Cell Renal Cell Carcinoma, Cellular Signalling. (2024) 124, 111425, 10.1016/j.cellsig.2024.111425, 39307376. 39307376

[25] Man Y.-N. , Sun Y. , Chen P. J. , Wu H. , and He M. L. , TAF1D Functions as a Novel Biomarker in Osteosarcoma, Journal of Cancer. (2023) 14, no. 11, 2051–2065, 10.7150/jca.85688, 37497412. 37497412 PMC10367927

[26] Cheng Q. , Zhao W. , Song X. , and Jin T. , Machine-Learning and scRNA-Seq-Based Diagnostic and Prognostic Models Illustrating Survival and Therapy Response of Lung Adenocarcinoma, Genes &amp; Immunity. (2024) 25, no. 5, 356–366, 10.1038/s41435-024-00289-0, 39075270. 39075270

[27] Ye J. , Wang J. , Tan L. , Yang S. , Xu L. , Wu X. , Deng H. , and Tan H. , Expression of Protein TARBP1 in Human Hepatocellular Carcinoma and Its Prognostic Significance, International Journal of Clinical and Experimental Pathology. (2015) 8, no. 8. PMC4583883 26464651

[28] Mo X. B. , Sun Y. H. , Wu L. F. , He P. , Cao R. R. , Lu X. , Zhang Y. H. , Deng F. Y. , and Lei S. F. , A Novel Long Non-Coding RNA, lnc-RNU12, Influences the T-Cell Cycle viac-JUNandCCNL2in Rheumatoid Arthritis, Rheumatology. (2023) 62, no. 5, 1955–1963, 10.1093/rheumatology/keac553. 36165706

[29] Liu W. , You W. , Lan Z. , Ren Y. , Gao S. , Li S. , Chen W. W. , Huang C. , Zeng Y. , Xiao N. , Wang Z. , Xie H. , Ma H. , Chen Y. , Wang G. , Chen C. , and Li H. , An Immune Cell Map of Human Lung Adenocarcinoma Development Reveals an Anti-Tumoral Role of the Tfh-Dependent Tertiary Lymphoid Structure, Cell Reports Medicine. (2024) 5, no. 3, 101448, 10.1016/j.xcrm.2024.101448, 38458196. 38458196 PMC10983046

[30] Shannon S. G. , The Characterization of the Tetratricopeptide Repeat Protein 13 in Mammals, 2012, MOspace.

[31] Alhebshi H. , Tian K. , Patnaik L. , Taylor R. , Bezecny P. , Hall C. , Muller P. A. J. , Safari N. , Creamer D. P. M. , Demonacos C. , Mutti L. , Bittar M. N. , and Krstic-Demonacos M. , Evaluation of the Role of p53 Tumour Suppressor Posttranslational Modifications and TTC5 Cofactor in Lung Cancer, International Journal of Molecular Sciences. (2021) 22, no. 24, 13198, 10.3390/ijms222413198, 34947995. 34947995 PMC8707832

[32] Chen C. , Li P. , Fan G. , Yang E. , Jing S. , Shi Y. , Gong Y. , Zhang L. , and Wang Z. , Role of TRIP13 in Human Cancer Development, Molecular Biology Reports. (2024) 51, no. 1, 10.1007/s11033-024-10012-x. 39436503

[33] Yu L. , Xiao Y. , Zhou X. , Wang J. , Chen S. , Peng T. , and Zhu X. , TRIP13 Interference Inhibits the Proliferation and Metastasis of Thyroid Cancer Cells Through Regulating TTC5/p53 Pathway and Epithelial-Mesenchymal Transition Related Genes Expression, Biomedicine &amp; Pharmacotherapy. (2019) 120, 109508, 10.1016/j.biopha.2019.109508, 2-s2.0-85073551012, 31648166. 31648166

