# Supplementary material for: Panomics Integration via Machine Learning Prioritizes TAF1D as a Therapeutic Vulnerability in Lung Adenocarcinoma
Source: Hum Mutat. 2026 Apr 11;2026:1816649. doi: 10.1155/humu/1816649 (PMC13069365; doi:10.1155/humu/1816649)

Supplementary Figure 1

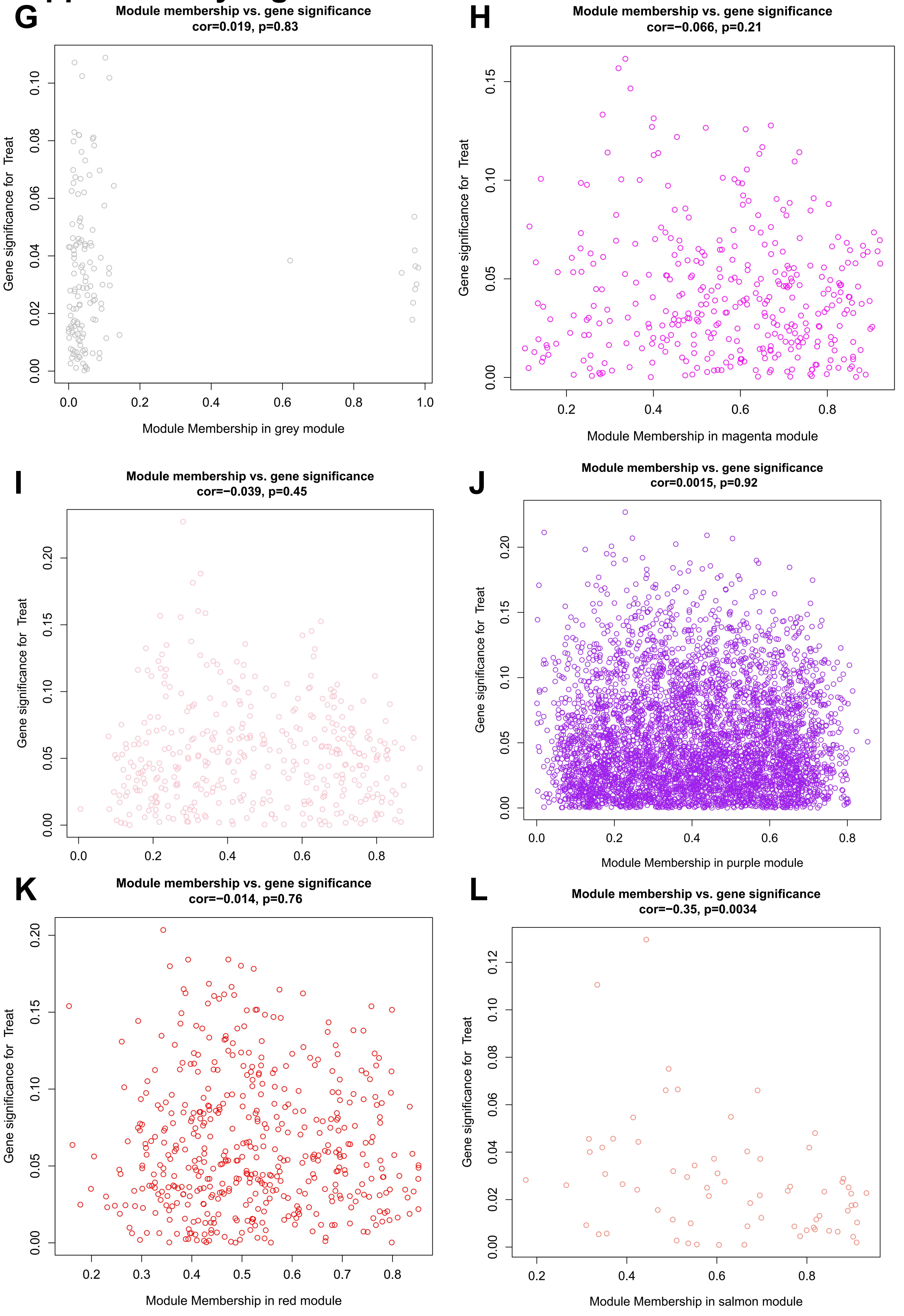

Supplement: Supplementary file 1 — Supporting Information 1 Figure S1: (A) Distribution of mean gene significance (GS) across modules. (B–N) Correlation between module membership (MM) and GS. (O) Protein–protein interaction (PPI) network. [file HUMU-2026-1816649-s001.zip › Supplementary Figure1 G-L.pdf]
