# Supplementary figures and images for: Panomics Integration via Machine Learning Prioritizes TAF1D as a Therapeutic Vulnerability in Lung Adenocarcinoma
Source: Hum Mutat. 2026 Apr 11;2026:1816649. doi: 10.1155/humu/1816649 (PMC13069365; doi:10.1155/humu/1816649)

## Supplementary Figure 1

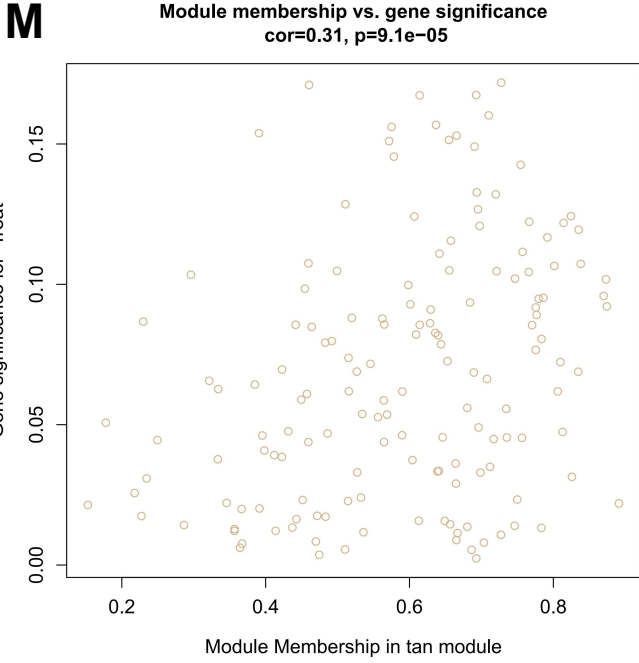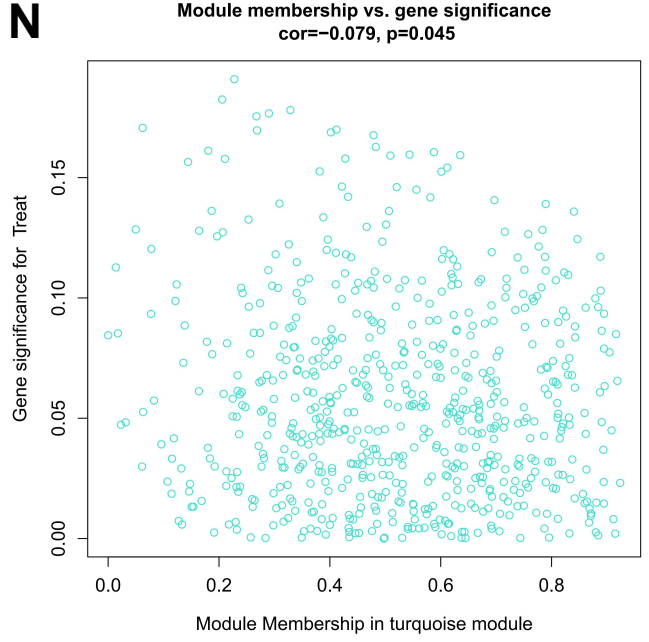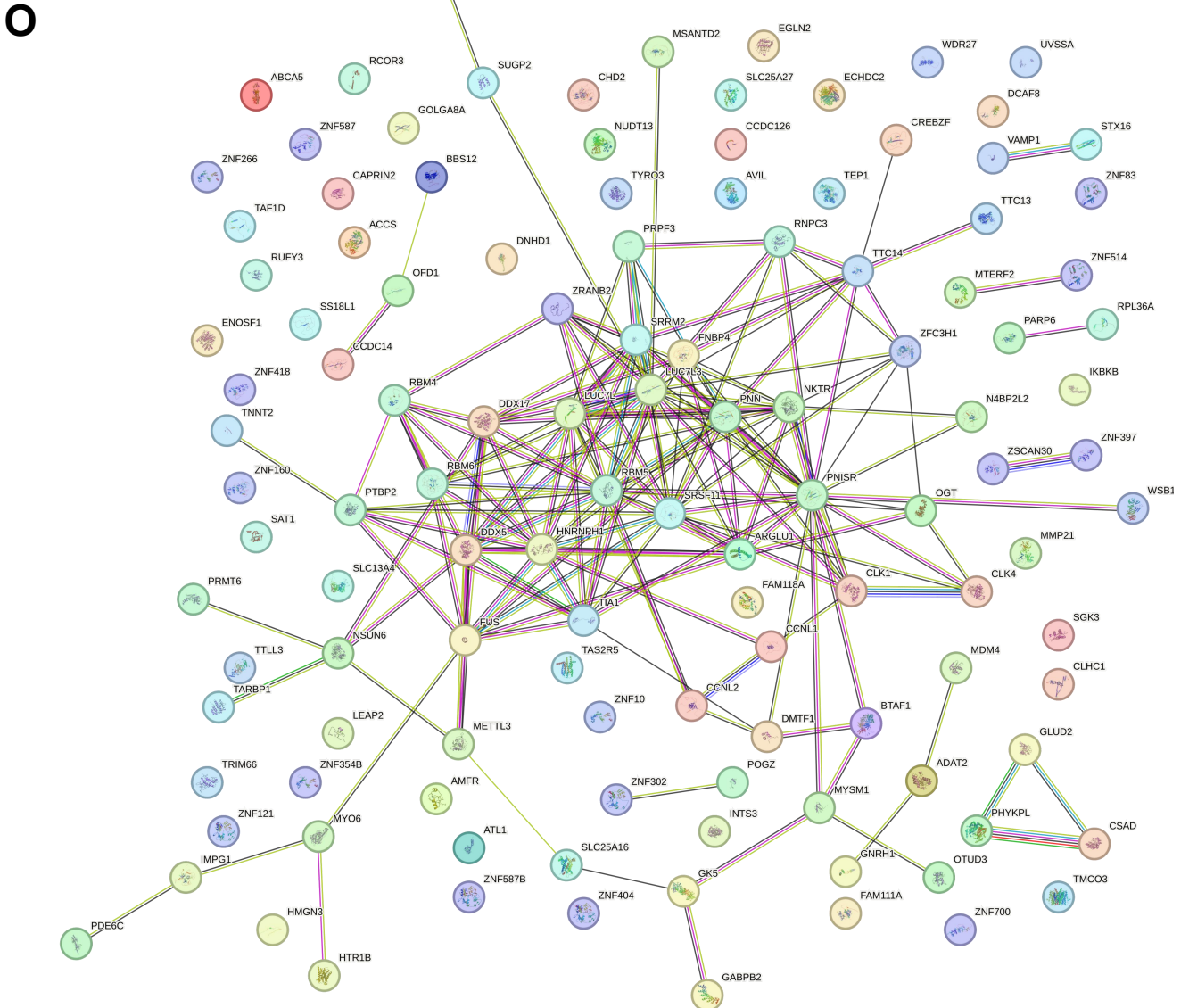

Supplement: Supplementary file 1 — Supporting Information 1 Figure S1: (A) Distribution of mean gene significance (GS) across modules. (B–N) Correlation between module membership (MM) and GS. (O) Protein–protein interaction (PPI) network. [file HUMU-2026-1816649-s001.zip › Supplementary Figure1 M-O.pdf]

# Supplementary Figure 1

**A**

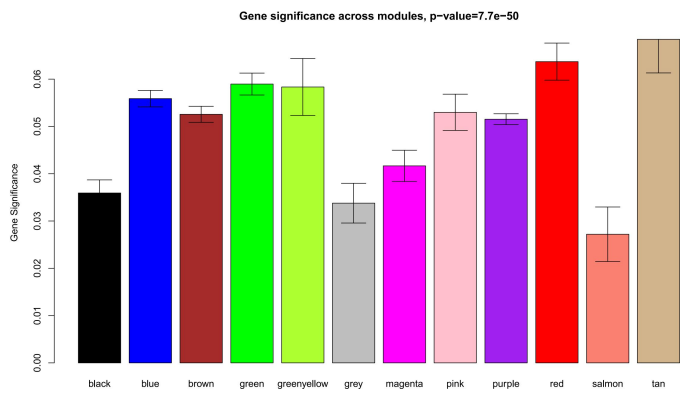

**B**

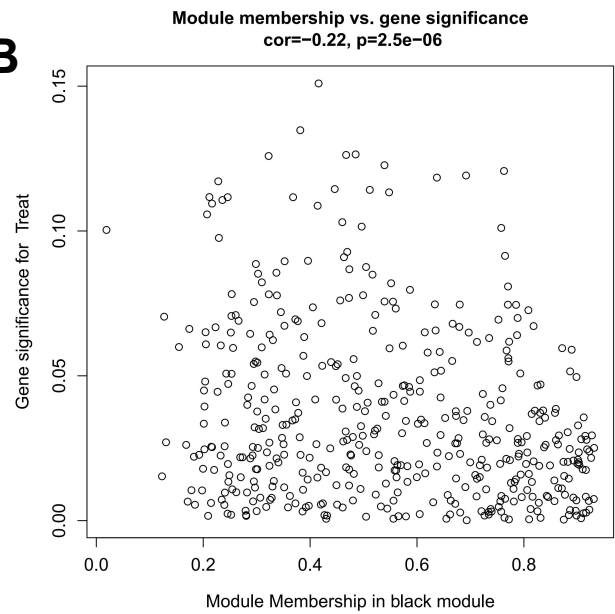

**C**

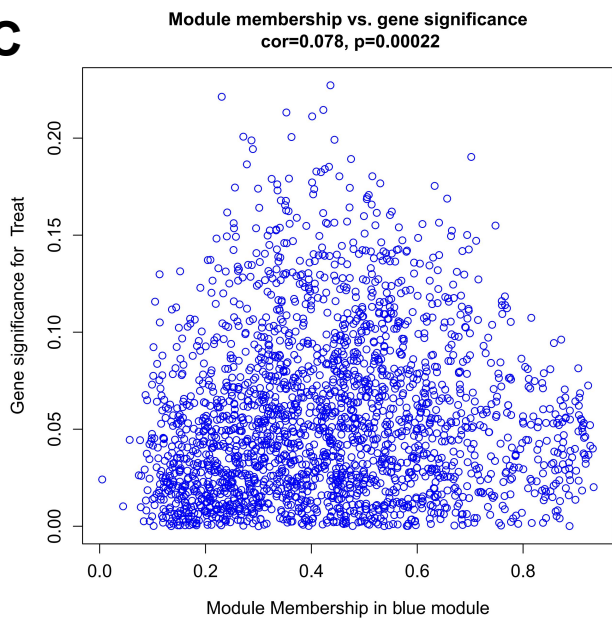

**D**

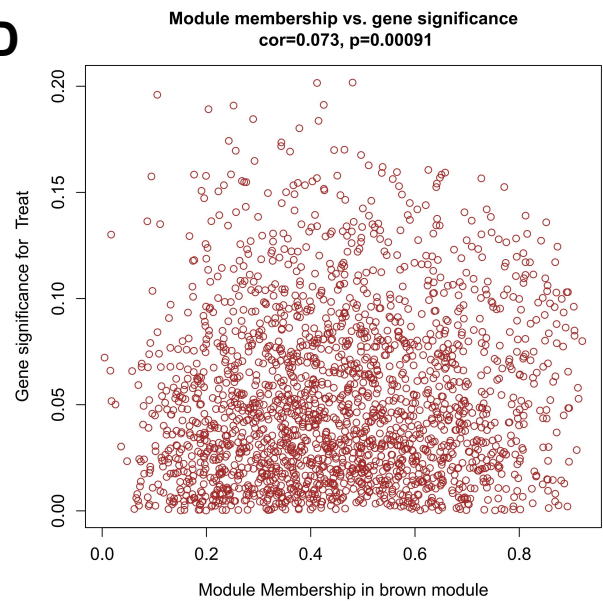

**E**

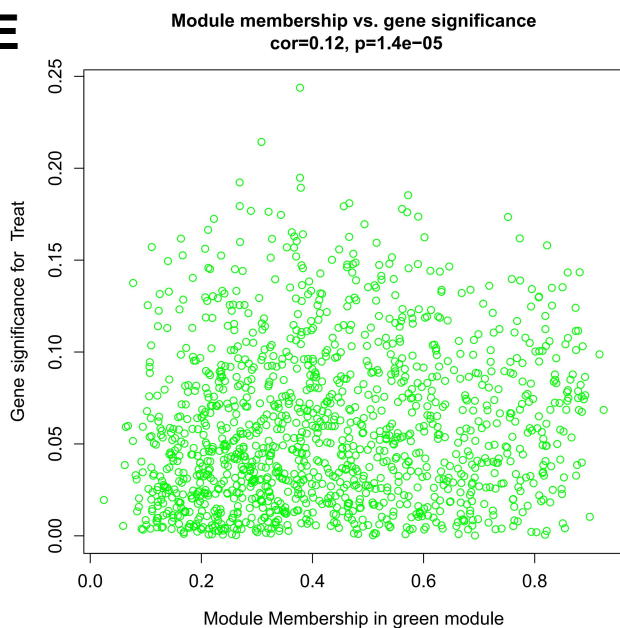

**F**

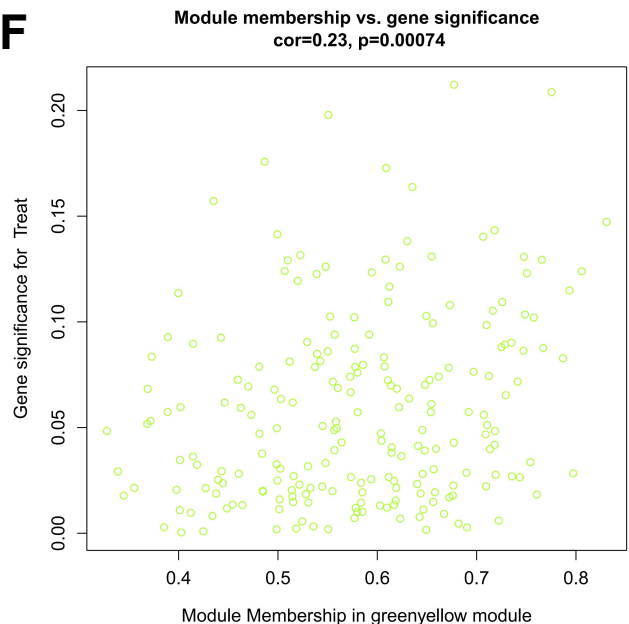

Supplement: Supplementary file 1 — Supporting Information 1 Figure S1: (A) Distribution of mean gene significance (GS) across modules. (B–N) Correlation between module membership (MM) and GS. (O) Protein–protein interaction (PPI) network. [file HUMU-2026-1816649-s001.zip › Supplementary Figure1 A-F.pdf]

Supplementary Figure 2

B

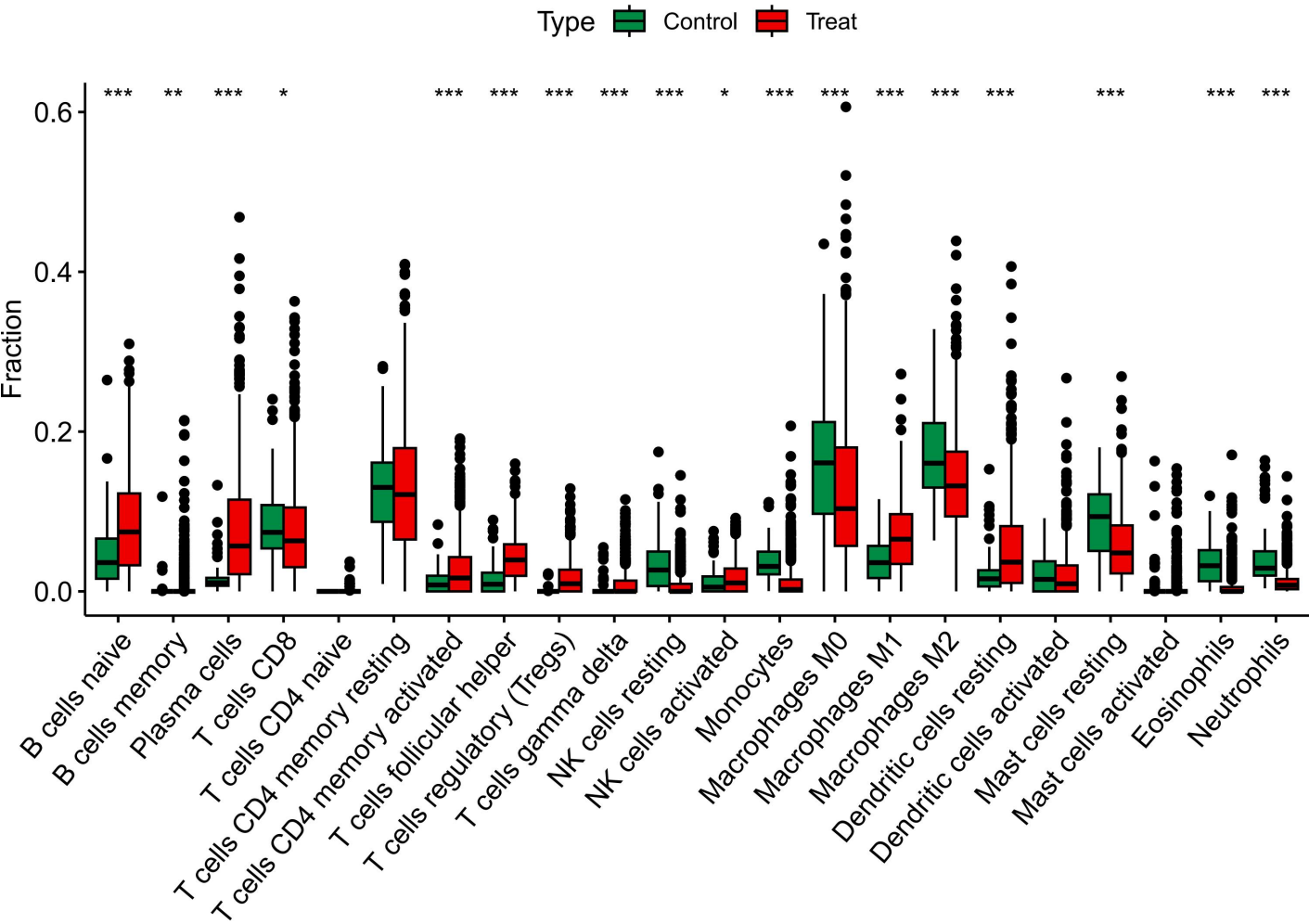

Supplement: Supplementary file 2 — Supporting Information 2 Figure S2: (A) Landscape of immune cell infiltration. (B) Synergistic correlation of immune cells. [file HUMU-2026-1816649-s002.zip › Supplementary Figure2 B.pdf]

# Supplementary Figure 2

A

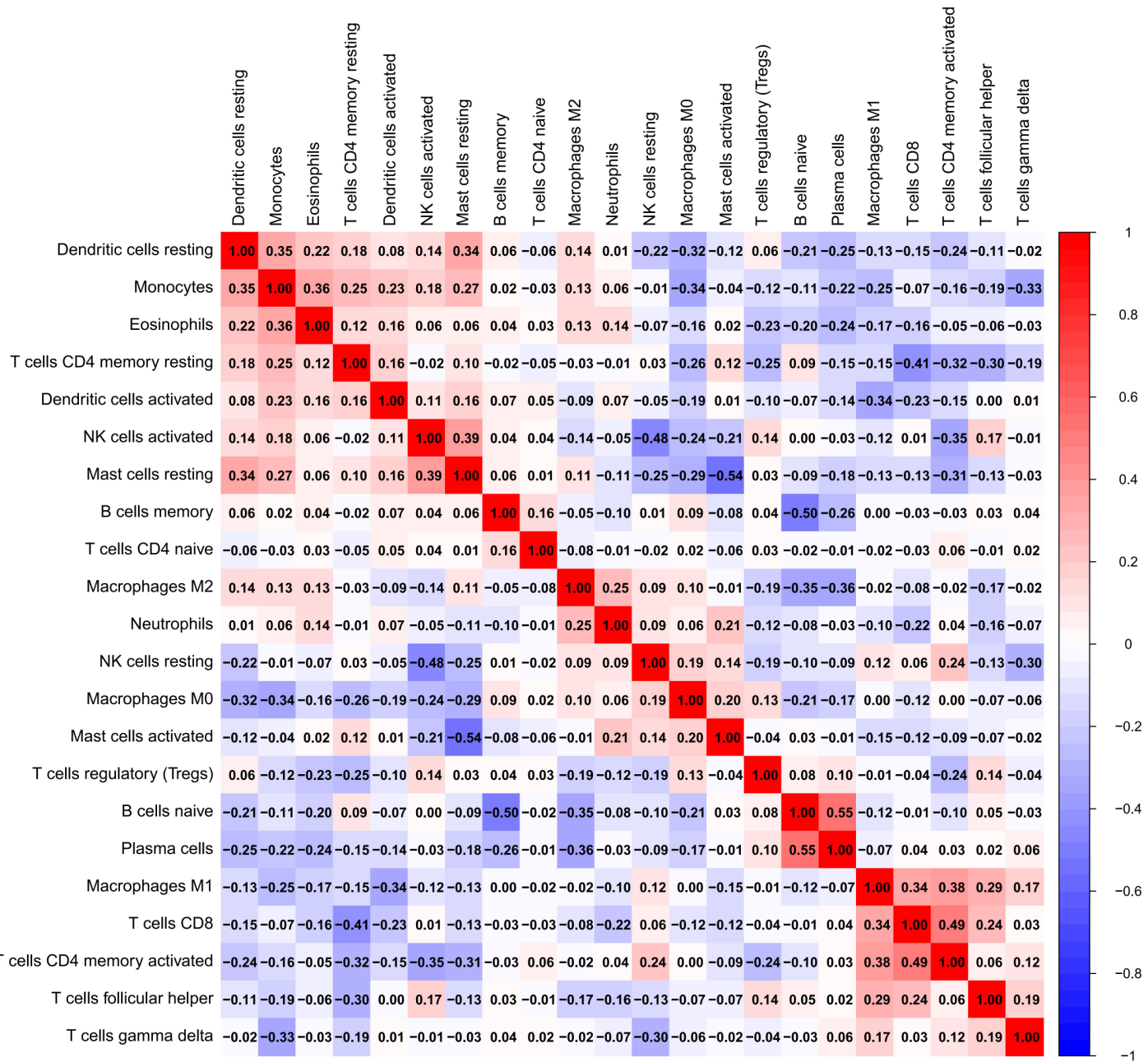

Supplement: Supplementary file 2 — Supporting Information 2 Figure S2: (A) Landscape of immune cell infiltration. (B) Synergistic correlation of immune cells. [file HUMU-2026-1816649-s002.zip › Supplementary Figure2 A.pdf]

# J

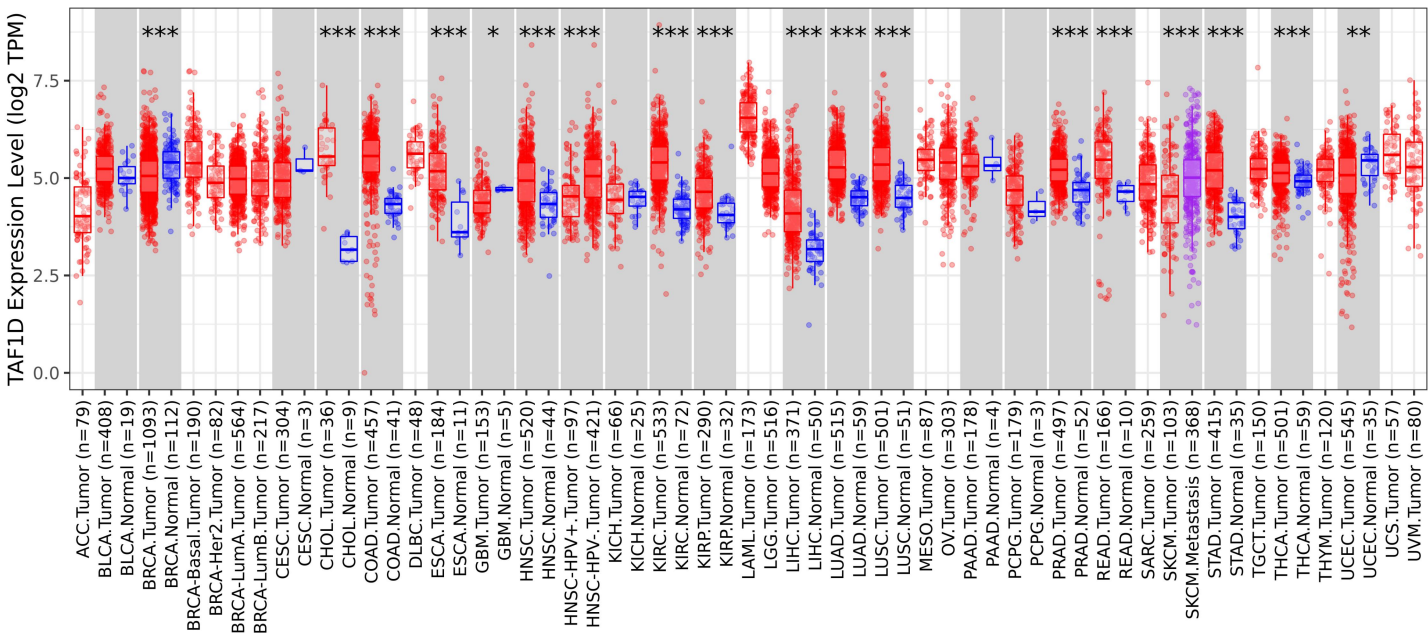

Supplement: Supplementary file 3 — Supporting Information 3 Figure S3: Clinical relevance analysis of TAF1D. (A) TAF1D expression levels in healthy human tissues. (B) TAF1D expression levels in the TCGA cohort. (C) TAF1D expression across different tumor stages. (D) TAF1D expression across different ethnicities. (E) TAF1D expression across different genders. (F) TAF1D expression across different age groups. (G) TAF1D expression across different smoking statuses. (H) TAF1D expression across different lymph node stages. (I) Prognostic impact of TAF1D high‐ and low‐expression groups. (J) TAF1D expression across 33 types of tumors. [file HUMU-2026-1816649-s006.zip › Supplementary Figure3 J.pdf]

# Supplementary Figure 3

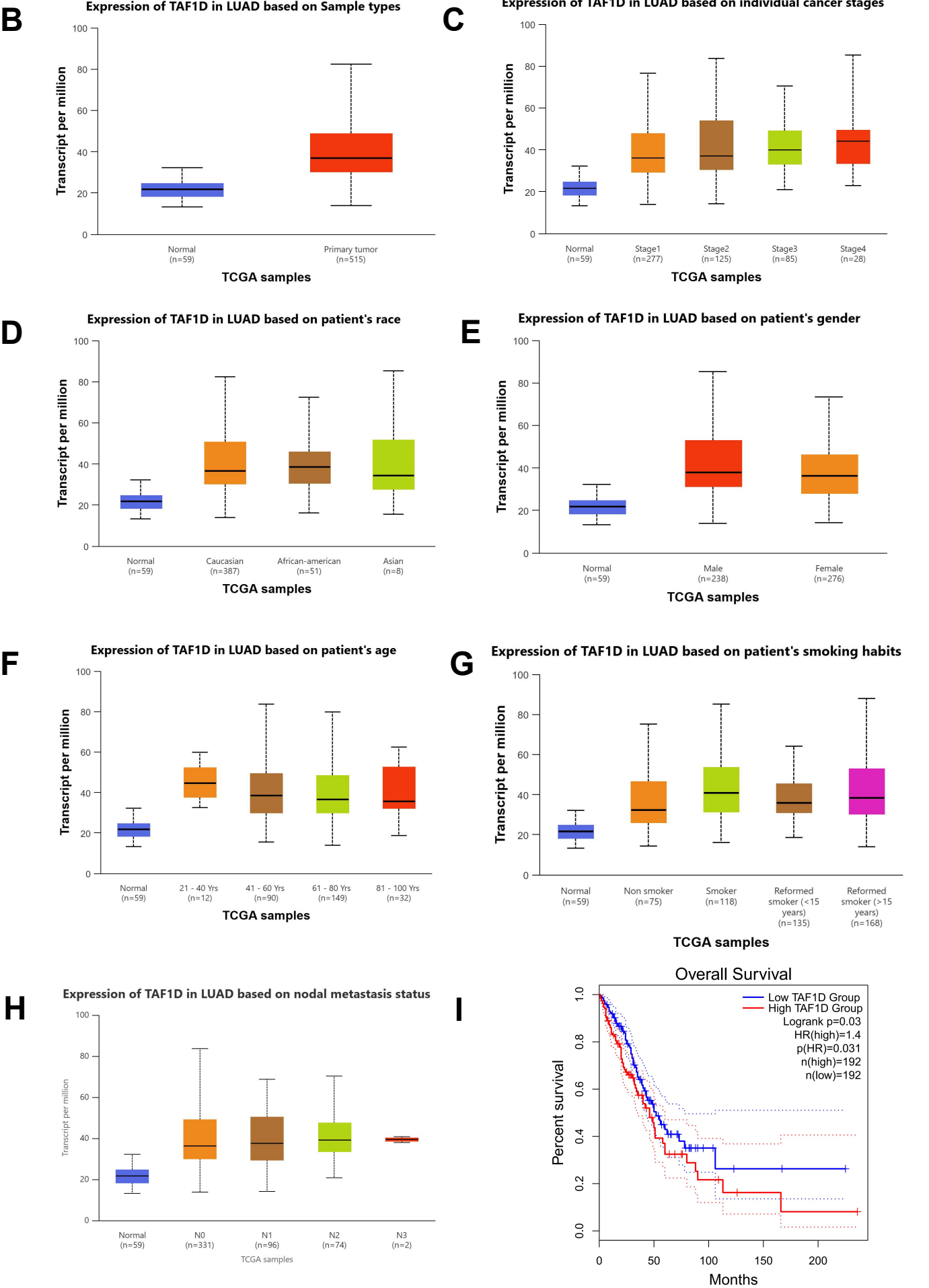

Supplement: Supplementary file 3 — Supporting Information 3 Figure S3: Clinical relevance analysis of TAF1D. (A) TAF1D expression levels in healthy human tissues. (B) TAF1D expression levels in the TCGA cohort. (C) TAF1D expression across different tumor stages. (D) TAF1D expression across different ethnicities. (E) TAF1D expression across different genders. (F) TAF1D expression across different age groups. (G) TAF1D expression across different smoking statuses. (H) TAF1D expression across different lymph node stages. (I) Prognostic impact of TAF1D high‐ and low‐expression groups. (J) TAF1D expression across 33 types of tumors. [file HUMU-2026-1816649-s006.zip › Supplementary Figure3 B-I.pdf]

# Supplementary Figure 3

A

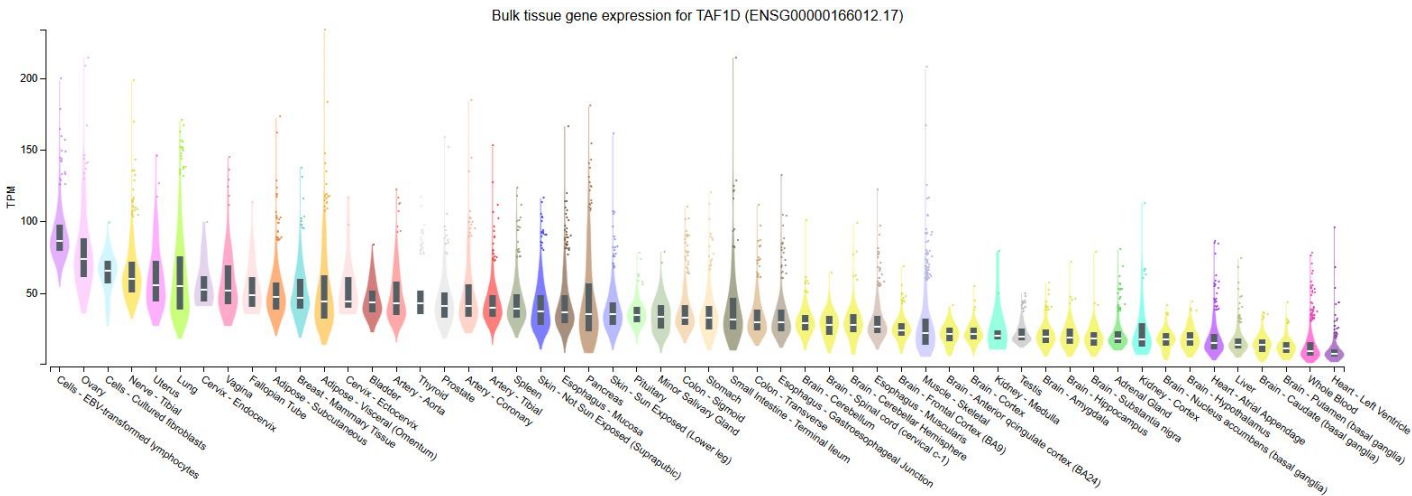

Supplement: Supplementary file 3 — Supporting Information 3 Figure S3: Clinical relevance analysis of TAF1D. (A) TAF1D expression levels in healthy human tissues. (B) TAF1D expression levels in the TCGA cohort. (C) TAF1D expression across different tumor stages. (D) TAF1D expression across different ethnicities. (E) TAF1D expression across different genders. (F) TAF1D expression across different age groups. (G) TAF1D expression across different smoking statuses. (H) TAF1D expression across different lymph node stages. (I) Prognostic impact of TAF1D high‐ and low‐expression groups. (J) TAF1D expression across 33 types of tumors. [file HUMU-2026-1816649-s006.zip › Supplementary Figure3 A.pdf]

# Supplementary Figure 4

A

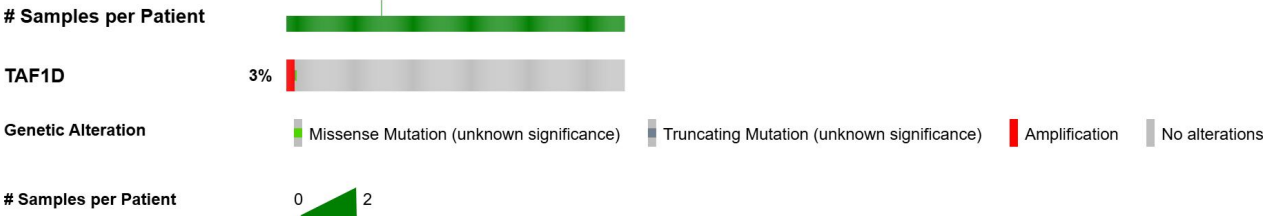

B

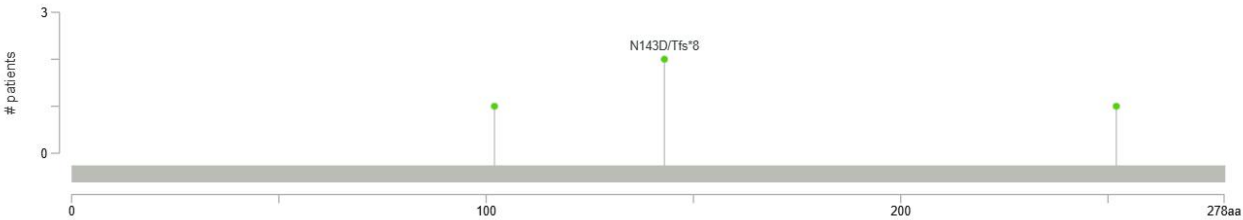

C

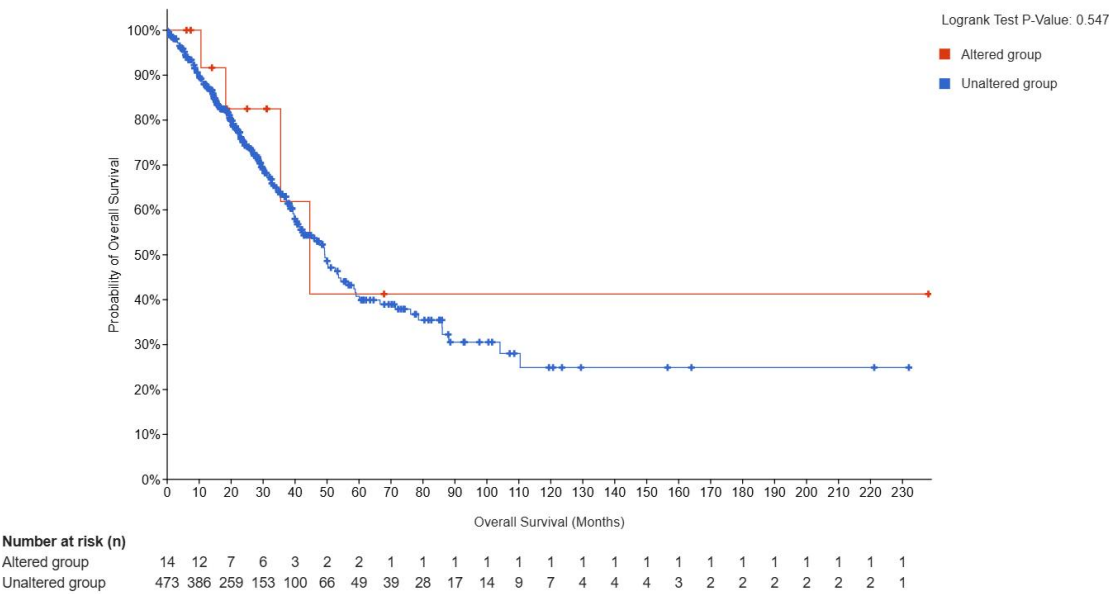

Supplement: Supplementary file 4 — Supporting Information 4 Figure S4: Genomic landscape and comutation pattern of TAF1D in lung adenocarcinoma. (A) OncoPrint illustrating the alteration frequency and mutation types of TAF1D in the TCGA‐LUAD cohort. (B) Lollipop plot showing the distribution of TAF1D somatic mutations across the protein sequence. (C) Kaplan–Meier overall survival analysis comparing patients with and without TAF1D alterations. [file HUMU-2026-1816649-s004.zip › Supplementary Figure4.pdf]

Supplementary Figure 5

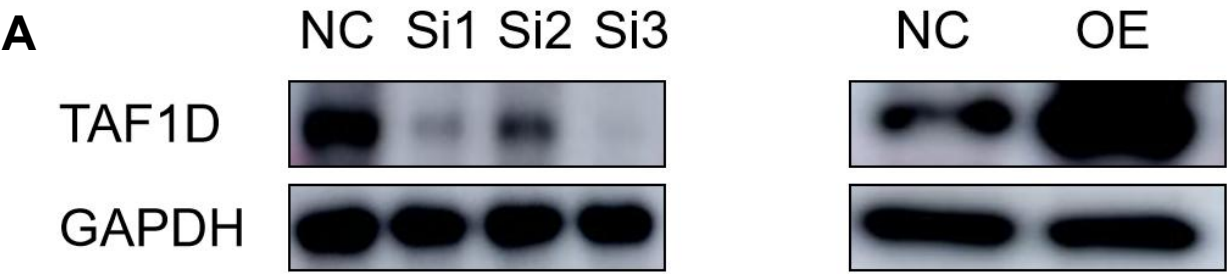

Supplement: Supplementary file 5 — Supporting Information 5 Figure S5: (A) Western blot images of overexpression of TAF1D (right panel) and downregulation efficiency (left panel). [file HUMU-2026-1816649-s005.zip › Supplementary Figure5.pdf]
